# From Devices to Systems: Integration Strategies for Micro‐Supercapacitors in Microsystems

**DOI:** 10.1002/advs.76806

**Published:** 2026-07-27

**Authors:** Zhuohao Liu, Gang Li, Kaiying Wang

**Affiliations:** ^1^ Institute of Energy Innovation College of Materials Science and Engineering & College of Mining Engineering Taiyuan University of Technology Taiyuan China; ^2^ Department of Microsystems University of South‐Eastern Norway Horten Norway

**Keywords:** integration, micro‐supercapacitor, microsystem, system‐oriented energy solution

## Abstract

Micro‐supercapacitors (MSCs) are emerging as indispensable energy units for integrated microsystems, where miniaturization, rapid power delivery, and long‐term stability must be achieved simultaneously. Despite substantial progress in electrode materials, device architectures, and microfabrication technologies, the field remains largely device‐centric, with advances still primarily evaluated by isolated electrochemical metrics rather than by practical system‐level functionality. Consequently, the central challenge is no longer simply to improve individual MSCs performance, but to establish a design logic that enables their effective integration into real microsystems. This Review redefines MSCs as system‐governed functional components and positions integration as the key variable linking application requirements, system constraints, device architectures, and material choices. The fundamental concepts of MSCs integration are first revisited from a microsystem perspective, including energy architectures, spatial and process constraints, and application‐relevant performance metrics. Monolithic and hybrid integration routes are then systematically compared, and a selection logic is established based on their trade‐offs in parasitic loss, coupling efficiency, fabrication compatibility, scalability, and functional evolvability. By shifting the field from device‐level optimization toward integration‐defined system design, this Review provides a conceptual framework and practical roadmap for advancing MSCs toward deployable microsystem energy solutions.

## Graphical abstract

Micro‐supercapacitors are evolving from device‐level energy storage toward system‐level functional units in integrated microsystems. This Review presents a system‐oriented design framework in which integration serves as the key link between application requirements, system constraints, device architectures, and material selection. Monolithic and hybrid strategies are compared to guide practical microsystem energy integration.

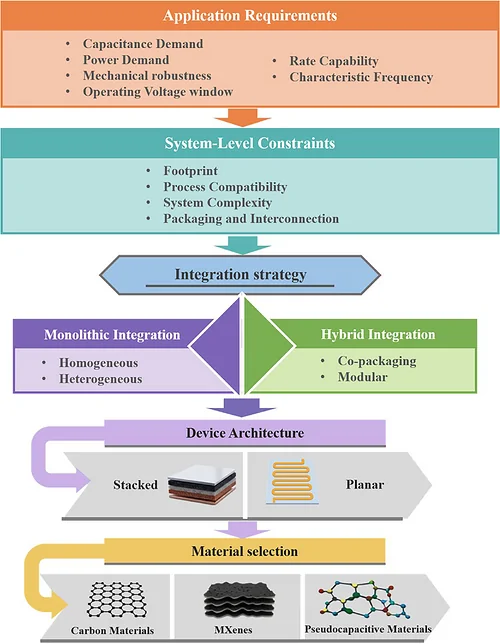

## 1 Introduction

The rapid advancement of micro/nanoelectromechanical systems (MEMS/NEMS), wearable electronics, implantable medical devices, and Internet of Things (IoT) platforms has created an increasing demand for compact, reliable, and efficient micro power sources [1, 2, 3, 4]. These emerging microsystems operate under stringent constraints in terms of footprint, mechanical compliance, and energy availability, rendering conventional energy storage technologies, particularly bulk batteries, unsuitable for direct integration into such systems [5, 6]. The inherent mismatch between traditional energy storage devices and microscale platforms, manifested in dimensions, mechanical rigidity, and fabrication incompatibility, has become a critical bottleneck limiting further miniaturization and functional integration [7, 8]. Consequently, the development of micro energy storage devices that can be seamlessly integrated into microsystems has emerged as a pivotal research direction. Among various candidates, MSCs have attracted considerable attention owing to their intrinsic microscale architecture, high power density, and long cycling stability, making them particularly suitable for energy buffering and transient power delivery in microsystems [9].

Over the past decade, extensive research efforts have led to a substantial body of review literature on MSCs. These studies can be broadly categorized into three main directions. First, materials‐focused reviews emphasize the development of advanced electrode materials, including carbon nanostructures [10], transition metal oxides [11], conductive polymers [12], and emerging two‐dimensional materials such as MXenes [13, 14], aiming to enhance capacitance, rate capability, and cycling stability. Second, device‐oriented investigations focus on structural design, covering planar interdigitated configurations, three‐dimensional architectures, and array‐based MSCs, with the objective of improving energy density and optimizing ion transport pathways [6, 15]. Third, more recent studies have begun to explore fabrication and integration techniques, including photolithography [16], printing [17, 18], and laser‐based processing [19, 20], highlighting progress toward the realization of microsystems.

Despite these advances, existing reviews remain largely centered on material innovation and device‐level performance, while the role of MSCs within complete microsystems has not been sufficiently addressed [21, 22]. In practical applications, MSCs do not operate as isolated devices but function as integral components within microsystem energy architectures, interacting with energy harvesting units, power management circuits, and functional modules [23, 24, 25, 26]. Under such conditions, their performance is governed not only by intrinsic material properties but also by system‐level constraints, including footprint limitations, fabrication compatibility, and functional scalability [27, 28, 29]. Moreover, integration strategies are often treated as downstream fabrication steps rather than as central design variables that dictate system architecture and performance. As a result, there remains a lack of a unified framework capable of systematically bridging material selection [30], device architecture [6, 15], integration strategies [31, 32], system constraints, and application requirements [4]. Addressing these limitations necessitates a shift toward a system‐oriented design perspective.

**FIGURE 1:**
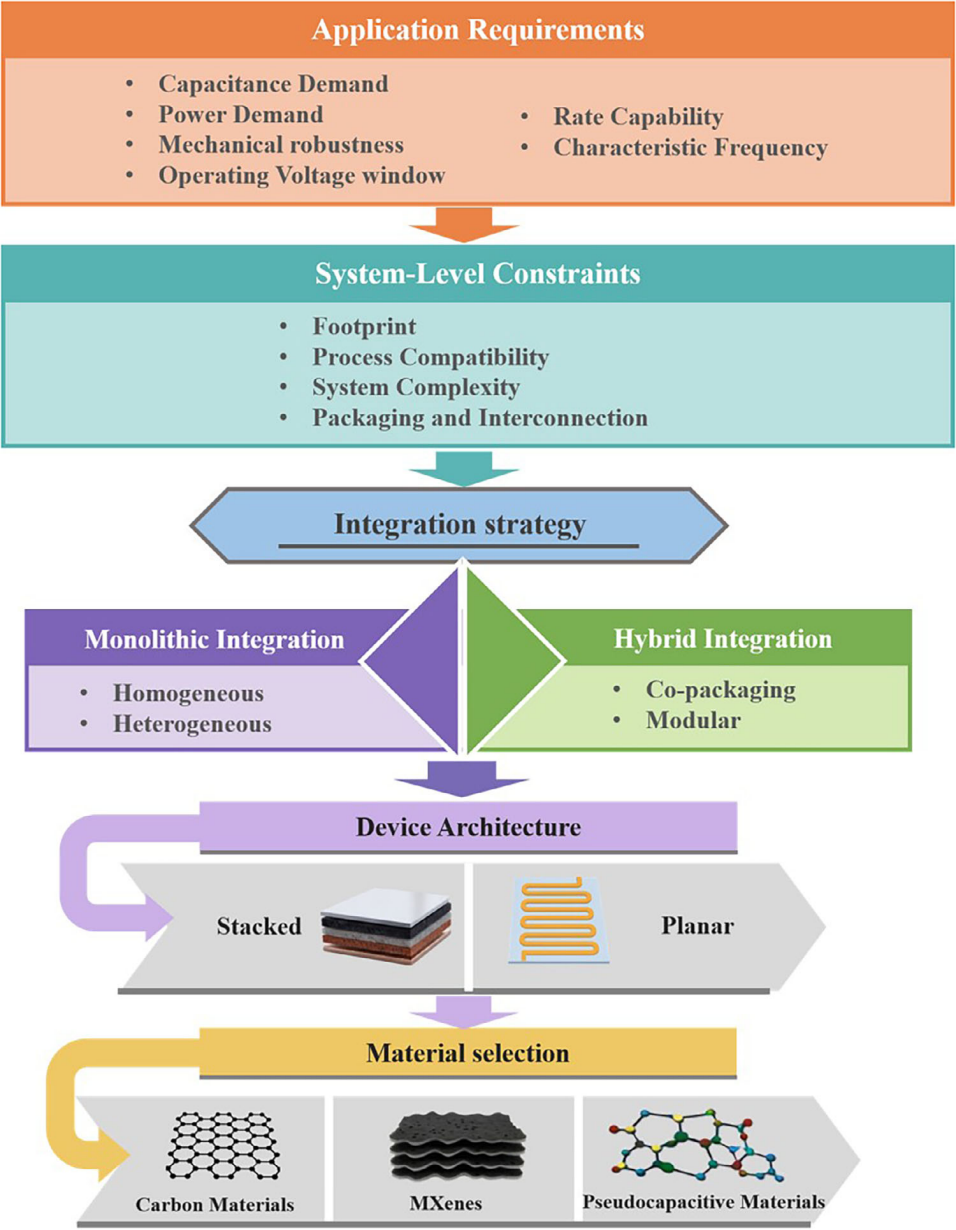
Framework for the integration design of MSCs within microsystems.

In response to this challenge, a system‐level, integration‐centered design framework is proposed, in which integration is treated as the governing variable that links application requirements to material selection. To address the lack of a unified system‐level design paradigm, it is necessary to establish a framework that explicitly connects these fragmented dimensions. As illustrated in Figure 1, this framework establishes a hierarchical design pathway spanning from application requirements to material selection, with integration strategies positioned as the central bridge connecting system constraints and device realization. Within this framework, application requirements define system‐level constraints, including footprint, functional scalability, and process compatibility limitations, which in turn guide the selection of integration strategies, such as monolithic or hybrid approaches [33]. These integration pathways subsequently determine the feasible device architectures and compatible material systems [32, 34, 35, 36]. In this context, integration is no longer viewed merely as a fabrication process but rather as a fundamental design dimension that governs the feasibility and performance of MSCs within microsystems. This framework thus shifts the conventional bottom‐up, material‐driven paradigm toward a top‐down, system‐driven design philosophy.

Building upon this framework, this review re‐examines MSCs from a system‐level perspective. Section 2 introduces the fundamental concepts of MSC integration, including microsystem energy architectures, system‐level constraints, and performance evaluation metrics. Sections 3 and 4 systematically compare monolithic and hybrid integration strategies in terms of structural characteristics, fabrication compatibility, scalability, and application suitability, while establishing a decision‐making logic for selecting appropriate integration approaches. Section 5 analyzes the functional roles of MSCs in representative applications, including wearable electronics, self‐powered sensing systems, pulsed energy harvesting, and AC line filtering, which highlights the interplay between integration strategy, system requirements, and device performance. Finally, Section 6 discusses future research directions from the perspective of system‐level design and practical deployment.

This review establishes a unified framework that bridges system‐level demands and device‐level implementation. By moving beyond conventional material‐ and device‐centric analyses, it provides a comprehensive design paradigm for MSCs toward microsystem integration. This system‐oriented perspective is expected to facilitate the rational design of micro‐scale energy storage devices and accelerate their transition from laboratory demonstrations to practical applications. In this review, the term “integrated microsystems” covers not only rigid on‐chip platforms based on semiconductor‐compatible substrates, but also flexible and wearable microsystems. In these systems, MSCs are spatially and functionally integrated with other components, such as sensing units, energy harvesters, or power management circuits.

## 2 Basic Concepts of MSCs for Integration

A microsystem is an intelligent machine that integrates multiple functional components, such as energy harvesters, energy storage units, sensors, and actuators. These components are situated on a shared or interconnected platform, and the overall size typically ranges from several millimeters to several centimeters [23, 24, 25]. Microsystems are designed to perform specific tasks (e.g., physiological monitoring [37], environmental sensing [38], actuation [39], or data transmission), wherein the performance and reliability of each component are interdependent. Microsystems include, but are not limited to, MEMS, wearable health monitoring devices, implantable medical devices, and Internet of Things (IoT) nodes. They can be fabricated on rigid semiconductor substrates (e.g., silicon [40], SOI) or on flexible/stretchable polymeric [41], paper‐based [42], or textile substrates [43]. Within this system, MSCs are not standalone devices but rather serve as the energy subsystem, providing functions such as power buffering, transient energy delivery, voltage regulation, or high‐frequency filtering. The integration scheme of MSCs (whether monolithic or hybrid) must be co‐designed with other functional units to satisfy system‐level constraints, including footprint, thermal budget, mechanical compliance, and scalability.

Although the term “MSCs” is widely used, a universally accepted quantitative boundary has not yet been established. Based on geometric dimensions, MSCs refer to energy storage elements in which the interdigital electrode width, interelectrode spacing, or device thickness is on the micrometer scale (typically 1–500 µm) and the overall footprint is less than 1 cm^2^. This definition is consistent with the pioneering work of Kyeremateng et al. [36] and is compatible with most microfabrication processes, including photolithography [16], laser direct writing [19], and printing techniques [17]. Conversely, devices with millimeter‐scale dimensions are still considered MSCs if their form factor and fabrication processes are compatible with microelectronics or flexible substrates, and if they are designed for integration into microsystems. It is important to stress that no rigid area cap is set. The essential role of MSCs lies in their integration into miniaturized electronic systems, not merely in their physical size. MSCs primarily operate on two energy storage mechanisms: electrochemical electrical double‐layer capacitance and pseudocapacitance. Electrical double‐layer capacitance arises from the electrostatic adsorption of electrolyte ions at the electrode/electrolyte interface, offering fast charge/discharge kinetics and excellent cycling stability, albeit with a limited specific capacitance. In contrast, pseudocapacitance originates from fast and reversible Faradaic reactions (e.g., redox reactions and intercalation processes) occurring at or near the electrode surface, which can deliver a higher capacitance but at the expense of slightly slower response kinetics and somewhat reduced cycling durability [5]. In practical research and applications, many MSCs electrodes combine both mechanisms to strike a balance between energy density and power density performance.

The term “flexible” also requires a clear definition. In this review, flexible MSCs are defined as devices capable of repeatedly bending, twisting, folding, and, if applicable, stretching, all while preserving structural integrity and adequate electrochemical performance. The evaluation of flexibility should be conducted by reporting the deformation modes, minimum bending radius or maximum applied strain, number of mechanical cycles, and the corresponding capacitance retention and ESR [44, 45, 46]. Therefore, flexible MSCs are not merely devices fabricated on soft substrates, rather, they are energy storage units that maintain good functionality under specified mechanical deformation conditions. Typical flexible substrates include polyimide [47], PET [46], PDMS [39], SEBS [41], paper [42], and elastomeric films [48], which enable the integration of MSCs into deformable microsystems.

### 2.1 Microsystem Energy Architecture and Functional Roles of MSCs

**FIGURE 2:**
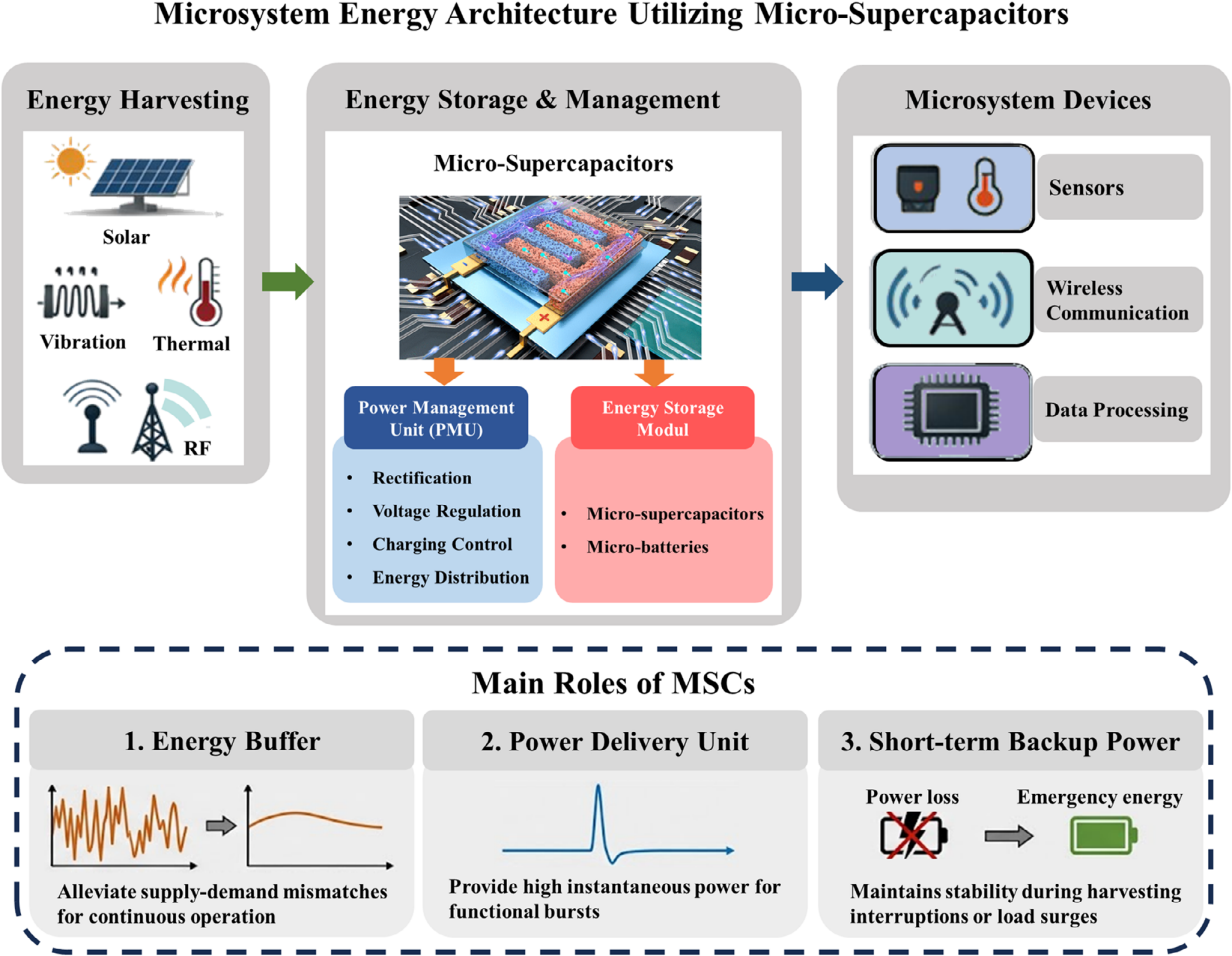
Energy architecture of microsystems and primary functions of MSCs. The figure is original, except for the diagram of MSCs, which is reproduced with permission from Ref. [15], Copyright 2022, American Chemical Society.

Understanding the system‐level energy architecture is a prerequisite for the rational design and integration of MSCs. As shown in Figure 2, a typical microsystem energy architecture consists of three interconnected subsystems: an energy harvesting module, an energy storage and management unit, and microsystem functional components. The energy harvesting module converts ambient energy sources (such as solar radiation, mechanical vibrations, thermal gradients, and radio frequency signals) into electrical energy [49, 50]. However, these energy sources are unstable, exhibiting low power density, intermittency, and temporal variability. For example, solar power output depends on illumination conditions [51, 52], vibrational energy varies with ambient motion [53], and radio frequency energy is typically weak and fluctuating [54]. Therefore, relying solely on harvested energy to directly power microsystems is often impractical.

To address this limitation, energy storage elements are incorporated to decouple the generation and consumption of power. Within energy storage, MSCs play a critical role due to their high‐power density and rapid charge/discharge capability. Compared with microbatteries (MBs), which are more suitable for long‐term energy supply, MSCs are better suited for handling transient energy flows and rapid load fluctuations [55, 56]. Therefore, MSCs are typically employed as intermediate energy buffers, storing energy from intermittent sources and releasing it upon demand. In numerous system architectures, MSCs are integrated with MBs to constitute hybrid energy storage systems, in which the battery ensures continuous energy delivery while the MSCs accommodate high‐power transient requirements [57, 58].

Beyond energy storage, the power management unit is essential to guarantee stable system operation. The power management unit orchestrates energy flow between the energy harvesting module, storage components, and functional loads via rectification, voltage regulation, charge control, and power allocation [59, 60, 61, 62]. The interplay between MSCs and the power management unit is of particular importance, given that it governs both the energy transfer efficiency and the system's dynamic responsiveness. Through mitigating voltage fluctuations, alleviating peak current demands, and stabilizing output signals, MSCs serve not merely as storage elements but as active dynamic regulators that augment system‐level performance [63, 64, 65]. However, MSCs are intrinsically plagued by severe self‐discharge behavior, arising from charge redistribution, leakage currents, and Faradaic side reactions at electrode/electrolyte interfaces, which causes a notable open‐circuit voltage decay and stored energy loss over short idle periods [66, 67, 68]. This inherent drawback makes active energy management not merely an optimization but a necessity, as passive standby is insufficient to maintain ready‐to‐use power. In this context, the power management unit must adopt adaptive strategies, such as voltage refresh pulses [69], or state‐of‐charge‐triggered reconditioning [70, 71], to compensate for self‐discharge losses, thereby preserving the MSCs’ terminal voltage within the operational window and ensuring reliable load supply despite the inherent leakage.

From a functional standpoint, MSCs generally assume three principal roles in microsystem energy architectures. First, MSCs serve as energy buffers that alleviate the discrepancy between intermittent power generation and dynamic load requirements, thus facilitating uninterrupted system operation [51, 55, 72]. Second, they operate as power delivery elements that supply high instantaneous power for functions including sensing, wireless communication, and signal processing [73]. Such operations frequently demand brief pulses of energy that surpass the inherent capabilities of standalone energy harvesters or MBs. Third, MSCs offer short‐term backup power to sustain system stability during transient disruptions in energy harvesting or abrupt load demand surges [74].

### 2.2 System‐Level Constraints for MSCs Integration

Despite these advantages, integrating MSCs into microsystems involves several critical constraints.

System footprint: One of the most important constraints is the limited footprint allocated to MSCs within the microsystem [16]. As device dimensions continue to shrink, the area allocated for energy storage becomes increasingly restricted. This necessitates the development of compact MSCs with high areal capacitance and efficient spatial utilization. Planar interdigitated designs are widely adopted due to their compatibility with microfabrication processes, but they typically offer limited energy density [6]. In contrast, three‐dimensional architectures can enhance energy storage by increasing the active surface area, although they introduce additional fabrication complexity and may present compatibility issues with existing manufacturing processes [75].

Fabrication compatibility: Fabrication compatibility constitutes an additional challenge, especially in the context of monolithic integration [76]. In this integration scheme, MSCs are directly fabricated onto the chip, necessitating rigorous compliance with fabrication limitations including thermal budgets, material compatibility, and lithographic resolution [29]. Electrode materials that deliver high performance typically require high‐temperature synthesis or intricate processing protocols, potentially conflicting with conventional semiconductor fabrication workflows [24, 77]. Consequently, a trade‐off must be negotiated between material performance and the practicality of integration. Hybrid integration, by comparison, permits MSCs to be fabricated independently and later assembled with the microsystem, thus affording enhanced flexibility in both materials selection and device optimization [78]. Nevertheless, this approach brings forth additional considerations, such as interconnect losses, packaging complexity, and long‐term reliability concerns.

Maintainability and scalability: Maintainability refers to whether MSCs support fault diagnosis or replacement, while scalability concerns the ability to flexibly adjust capacity by adding or removing units. For non‐replaceable scenarios such as implantable devices, MSCs are required to possess extremely high stability and packaging integrity [79, 80], whereas for wearable or modular systems, support for module replacement or capacity expansion is needed [81, 82]. Monolithic integration fixes the capacity and limits post‐fabrication adjustments. By contrast, hybrid integration, particularly in modular configurations, offers greater flexibility in maintenance and scalability [78]. Therefore, integration strategies must be comprehensively balanced against application requirements during the early stages of system design.

### 2.3 System‐Level Performance Evaluation of MSCs

These system‐level constraints underscore that the performance of MSCs must not be assessed solely on the basis of inherent material characteristics or device‐scale figures of merit. Rather, their efficacy should be evaluated in the broader context of the entire microsystem, accounting for interactions with other components and the prevailing operational conditions [4].

Performance metrics under spatial constraints: In on‐chip microsystems and flexible wearable electronics, the areal footprint or volumetric footprint of the device constitutes a primary constraint. Consequently, areal capacitance (mF·cm^−^^2^) and areal energy density (µWh·cm^−^^2^) take precedence over conventional gravimetric capacitance as the critical metrics for assessing energy storage efficacy. A high areal capacitance signifies enhanced energy storage capacity within a constrained chip footprint, directly impacting the system's operational runtime and endurance [83].

Stability criteria under dynamic environments: For wearable and epidermal electronics, the coupling of mechanical flexibility and electrochemical stability is of critical importance. Evaluation criteria include not only the capacitance value under static conditions but also emphasize capacitance retention under repeated and complex deformations (e.g., bending, stretching, twisting), as well as reliability after long‐term cyclic deformation [84]. For implantable medical devices, the primary criteria shift to biocompatibility, long‐term stability in biofluidic environments, and ultra‐low leakage current after encapsulation, ensuring system safety and long‐term operational reliability [85, 86].

Performance metrics for high‐frequency dynamics: For on‐chip AC line filtering applications, energy density ceases to be a primary concern, giving way to frequency response behavior. Under such circumstances, the critical performance metric becomes the equivalent series resistance (ESR), which must be maintained at an ultra‐low level to facilitate fast charge–discharge kinetics [87, 88]. For 60 Hz ripple filtering applications, the RC time constant (τ_0_ = 1/(2πf_0_)) is required to be below 8.3 ms [88]. Furthermore, the filtering performance of MSCs approaches that of an ideal capacitor as their phase angle at 120 Hz draws nearer to −90° [89].

Critical metrics for energy management: In self‐powered systems and energy harvesting scenarios, wherein MSCs function as energy buffering and dispatching nodes, leakage current and Coulombic efficiency emerge as pivotal system‐level parameters. A low leakage current guarantees that the minute amounts of harvested energy are effectively accumulated rather than lost. A high Coulombic efficiency signifies minimal energy dissipation during the storage and release cycle, a characteristic of paramount importance for microsystems operating under stringent energy budgets [16, 90].

Taken together, the design and evaluation of MSCs for integrated microsystems must be fundamentally reframed from a system‐level perspective. Rather than being governed solely by intrinsic material properties or isolated device metrics, the performance and functionality of MSCs are co‐determined by their roles within the microsystem energy architecture, the constraints imposed by footprint, fabrication compatibility, and scalability, as well as the application‐specific performance criteria. These interdependent factors collectively define a multi‐dimensional design space, in which MSCs operate as system‐coupled functional units rather than standalone energy storage devices. Within this context, the choice of integration strategy emerges as the critical link that translates system‐level requirements into practical device realization. It not only determines the physical configuration and coupling mode of MSCs with other functional components, but also constrains the accessible material systems, fabrication processes, and ultimately the achievable system performance. Therefore, to bridge the gap between system demands and device implementation, the following sections will systematically examine the major integration approaches for MSCs, with a particular focus on monolithic and hybrid strategies, and their respective trade‐offs in enabling efficient and scalable microsystem integration.

## 3 Monolithic Integration

Monolithic integration represents a critical pathway for achieving miniaturization and high integration density in MSCs, entailing the simultaneous fabrication of energy storage units and other functional modules on a single chip substrate. Within the system‐oriented framework proposed in this review, monolithic integration corresponds to the integration mode in which the highest degree of intrinsic coupling is pursued to satisfy stringent system‐level constraints such as footprint minimization, parasitic‐loss suppression, and high‐frequency or high‐voltage operation. According to functional attributes, monolithic integration is divisible into two distinct modalities: monolithic homogeneous integration and monolithic heterogeneous integration [16, 91]. Monolithic homogeneous integration involves the arrangement of multiple identical MSC storage cells into an array on the same chip, whereby structural and process uniformity guarantees parametric consistency among individual units, thus facilitating linear expansion of voltage or capacity. This approach stresses “structural uniformity,” mandating a high degree of congruence in electrode dimensions, material dispersion, and microstructural morphology to mitigate cell‐to‐cell performance variations [16]. In contrast, monolithic heterogeneous integration involves the simultaneous integration of disparate functional units on the same chip, for instance combining MSCs with sensors, energy harvesters, or signal processing modules, with the critical aspect being interfacial compatibility and performance coupling between distinct physical blocks. Monolithic heterogeneous integration prioritizes “parametric coordination,” which entails guaranteeing that all functional constituents operate harmoniously with respect to voltage levels, current handling, and transient response, ultimately enabling the realization of self‐powered, sensor‐integrated multifunctional microsystems [91, 92]. Accordingly, this section focuses on how intrinsic coupling is translated into system‐level performance advantages under different functional demands, thereby linking the proposed integration logic to the practical realization of microsystem functions.

### 3.1 Monolithic Homogeneous Integration

Monolithic homogeneous integration is not simply the fabrication of multiple MSC units on a single substrate, rather, it enables predictable and scalable electrical behavior at the array level via the co‐design of structure and fabrication processes [93]. Within this approach, system performance is governed by the cooperative response between units, as opposed to the optimized performance of a single device [94]. Hence, structural uniformity constitutes an essential precondition for the validity of monolithic homogeneous integration [95]. At the system level, this consistency manifests in three main areas. First, the homogeneity of electrode geometric parameters (e.g., line width, spacing, and thickness) dictates the ion transport paths and the ESR distribution [34]. Second, the mass loading and microstructure of the active material influence the uniformity of the capacitive response [95]. Third, the electrolyte coverage and interfacial contact conditions govern the homogeneity of charge transfer kinetics [16]. Together, these factors dictate the synchronicity of the cells in the array during charging and discharging, and directly impact the voltage balancing upon series integration. In series‐connected arrays operating at high voltage, small variations between cells become magnified, resulting in local overcharge or performance decay. Hence, in contrast to optimizing single‐device performance, monolithic homogeneous integration prioritizes the mitigation of cell‐to‐cell variation [89].

**FIGURE 3:**
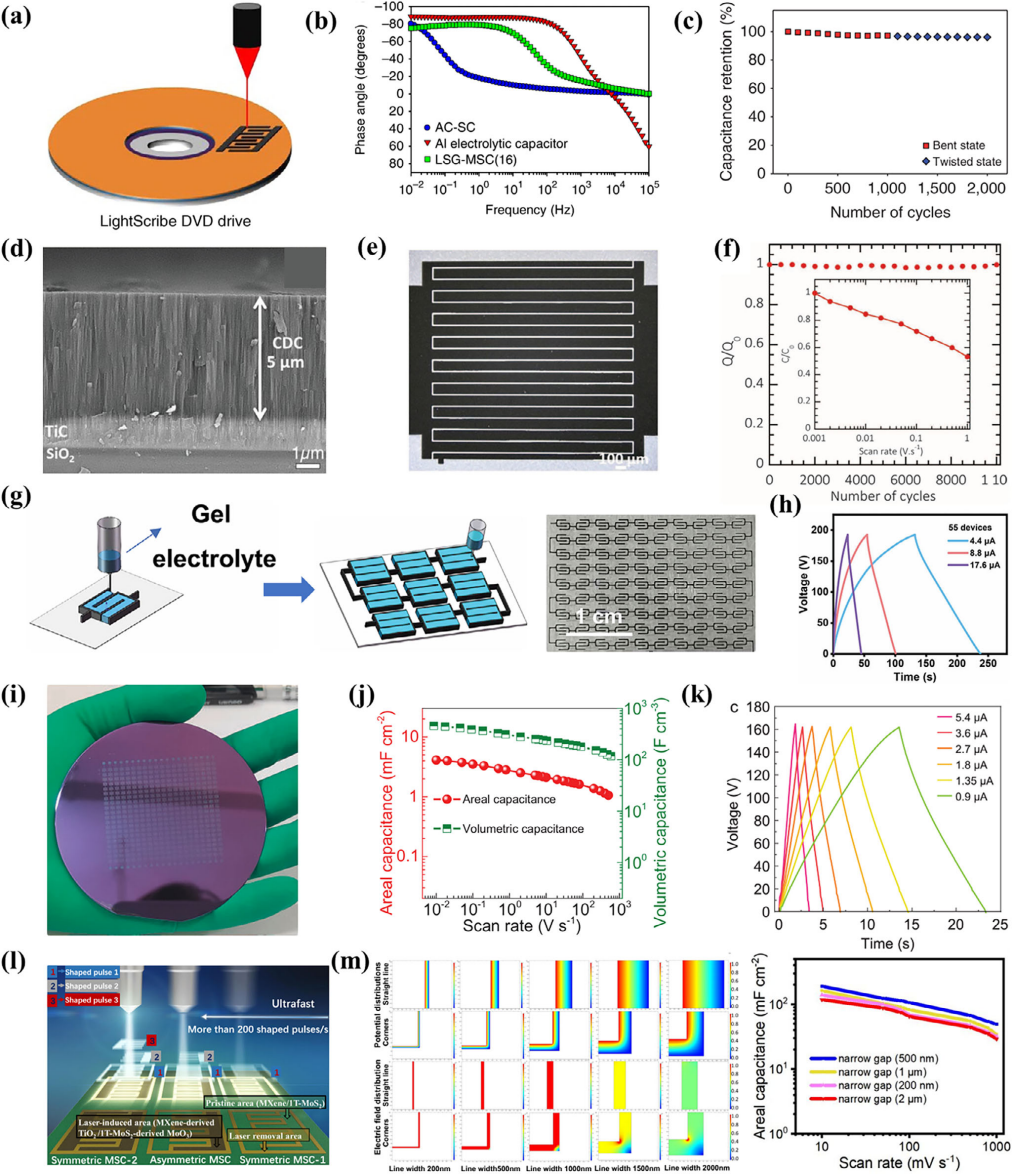
Fabrication and investigation of monolithically homogeneous integrated MSCs. (a) Schematic illustration of the fabrication of graphene‑based MSCs on a DVD. (b) Bode plots of graphene MSCs compared with commercial AC‑SC and aluminum electrolytic capacitors. (c) Stability of graphene MSCs tested under bending and twisting conditions. Reproduced with permission from Ref. [47], Copyright 2013, Springer Nature. (d) SEM image of a silicon‑based TiC/CDC thin film. (e) MSC with an electrode spacing of 15 µm. (f) Cycling stability of the Si/TiC/CDC electrode. Reproduced with permission from Ref. [77], Copyright 2016, The American Association for the Advancement of Science. (g) Graphene‑based MSCs fabricated via 3D printing. (h) Voltage window of monolithically integrated MSCs. Reproduced with permission from Ref. [96], Copyright 2024, Wiley‐VCH. (i) Hundreds of MSCs integrated on a Si/SiO_2_ substrate. (j) Rate capability of the MSCs. (k) Charge–discharge curves of monolithically integrated MSCs at 162 V. Reproduced with permission from Ref. [34], Copyright 2022, The Author(s). (l) Schematic diagram of MSC fabrication using femtosecond laser processing. (m) Investigation of electric field distribution in MSCs with varying line widths and actual MSCs capacitance using COMSOL software. Reproduced with permission from Ref. [97], Copyright 2023, The Author(s).

Under the constraints imposed by the above structural consistency requirements, the choice of material system emerges as a decisive element for the viability of monolithic homogeneous integration. In particular, carbon‐based materials (e.g., carbon nanotubes [24], carbide‐derived carbon [77], graphene [42], etc.) and MXene [16] have become the most prevalent choices at present, owing to their excellent electrical conductivity and favorable process compatibility. First, these materials typically feature either uninterrupted *π*‐conjugated frameworks or metallic‐type conductivity, which facilitate the establishment of low‐resistivity conduction pathways in microscale electrodes, markedly lower ESR, and improve the temporal synchronization of responses across the array elements. Second, their thin‐film morphology and versatile deposition methods (e.g., vapor‐phase growth or in situ conversion) render them directly compatible with planar nanofabrication techniques [77]. Additionally, carbon‐based materials exhibit favorable attributes in terms of thickness tunability and surface chemical robustness, thereby supporting the preservation of consistent charge‐storage performance over extended array dimensions. As shown in Figure 3a, El‐Kady et al. [47] employed laser reduction of graphene oxide to rapidly fabricate more than 100 MSCs on a flexible substrate, the resulting devices demonstrated a fast response time of approximately 19 ms and excellent bending stability (Figure 3b,c). Nevertheless, this approach depends on localized energy deposition, and the non‐uniformity in pattern boundaries and conductive networks tends to induce variations in resistance and capacitance across cells, thus constraining the consistency of the array. By comparison, photolithographic approaches derived from silicon processing offer micrometer‐scale or even finer pattern control. As shown in Figure 3d, Huang et al. [77] prepared CDC electrodes through TiC chlorination, realizing an interdigitated architecture with an electrode gap of roughly 15 µm. They integrated approximately 40 MSC units at the wafer scale, and the capacitance exhibited virtually no degradation after 10 000 cycles (Figure 3e,f). These methods provide notable benefits in array consistency and replicability, rendering them particularly suitable for high‐density monolithic integration, nevertheless, they often involve high‐temperature steps such as chlorination, which impose limitations in terms of thermal budget and material compatibility.

Once the material system is selected, the fabrication strategy subsequently governs the feasibility of achieving structural consistency within monolithic integration during practical manufacturing processes. Broadly speaking, prevailing fabrication methodologies can be categorized into techniques such as printing [98, 99], photolithography [34], and laser direct writing [97, 100], where the key difference resides in the patterning approach, thereby impacting the consistency across MSC arrays. As shown in Figure 3g, Zhang et al. [96] fabricated an array comprising 180 MSC units on a single substrate via 3D printing of graphene electrodes, the resulting device demonstrated favorable rate capability and an output voltage exceeding 190 V (Figure 3h), highlighting its potential for high‐voltage integration. Nevertheless, this approach is limited by ink rheological properties and deposition accuracy, leading to compromised pattern edge definition and thickness uniformity, which presents ongoing challenges for the fabrication of high‐uniformity arrays. By comparison, hybrid approaches that integrate photolithographic patterning with low‐temperature material deposition such as MXene spraying or gel electrolyte printing have achieved a pragmatic balance between patterning resolution and process compatibility. As shown in Figure 3i, Wang et al. [34] integrated 400 electrochemically isolated MSC units on a 3‐inch Si/SiO_2_ substrate and achieved a stable output exceeding 160 V while maintaining a high areal capacitance of 4.1 mF cm^−^^2^ (Figure 3j,k), indicating that the combination of high‐precision patterning and low‐temperature deposition represents an effective strategy for enhancing uniformity. Additionally, as shown in Figure 3l, femtosecond laser fabrication through optimization of energy distribution and processing dimensions enables the concurrent construction of symmetric and asymmetric MSCs on a monolithic thin film. When coupled with finite element simulations to refine electrode spacing (Figure 3m), this method further improves device uniformity and design flexibility, nevertheless, its reliability for large‐scale production still requires further verification [97]. Table 1 summarizes the key parameters of recent representative works, including processing temperature, device integration density, areal output voltage, and areal specific capacitance, further reflecting the performance characteristics of monolithically integrated MSCs across different material systems and fabrication routes.

**TABLE 1:** Comparison of parameters for monolithic integrated MSCs based on different material systems.

Electrode material	Process and temperature	Integration density (cells/cm^2^)	Maximum output voltage (V/cm^2^)	Areal capacitance (mF/cm^2^)	Characteristic frequency (f_0_/Hz)	Ref.
MXene	Photolithography, spray printing	210	555	2.3 (at 0.15 mA cm^−^^2^)		[16]
nAC	Inkjet printing, vacuum drying (50°C)	54.9	65.9	0.1275 (at 1 mV s^−^^1^)		[23]
MXene /CNT	Photolithography, annealing (120°C, 900°C)	107/8‐inch wafer				[24]
MXene	Photolithography, spray printing	28	75.6	4.1 (at 10 mV s^−^^1^)	2153 Hz (at −45°)	[34]
GR /PEDOT:PSS	Electrodeposition (room temp), femtosecond laser	80		5.2 (at 120 Hz)	1211 Hz (at −45°)	[89]
EG	3D printing	16	56	4900 (at 1 mV s^−^^1^)		[96]
MXene‐IL	Laser direct writing, heat treatment (360°C)			44.6 (at 0.3 mA cm^−^^2^)		[100]
PEDOT:PSS/GR	DIW (substrate temp 80°C), femtosecond laser, inkjet printing	100/(2.4 × 3.4)	160/(2.4 × 3.4)	> 60 (at 1 V s^−^^1^)		[42]
MXene	Photolithography, spray coating at 70°C			0.153 (at 1 V s^−^^1^)	5600 Hz (at −45°)	[40]
CNT	Screen printing, pre‐stretching			13.5 (at 3.6 mA cm^−^^3^)		[101]
1T‐MoS_2_/rGO	Inkjet printing, vacuum drying (50°C)			9.04 (at 20 mV s^−^^1^)		[99]
PEDOT:PSS/GR	DIW (substrate temp 80°C), laser	400/(12.5 × 7.5)	640/(12.5 × 7.5)			[102]

With the concerted underpinning of the aforesaid material systems and processing pathways, monolithic homogeneous integrated MSCs manifest array‐level performance strengths centered upon high power delivery. First, the percolating conductive pathways formed within carbon‐based electrodes, together with the precisely tailored microscale interdigital geometry, confer low ESR upon the devices, which facilitates fast charging/discharging kinetics and yields response times on the order of milliseconds [96]. Second, the concurrence of diminished ESR and abbreviated ion transport lengths jointly bestows high power density upon MSCs, providing a pronounced edge in applications involving pulsed energy delivery and momentary power bursts [42]. Moreover, as all constituent cells are concurrently constructed on a common substrate through an identical fabrication protocol, the resulting geometric and compositional homogeneity reduces cell‐to‐cell variation and promotes synchronized array behavior throughout charge/discharge operations, a characteristic that is especially advantageous for maintaining voltage equilibration and steady output in series‐connected configurations [16].

Although the considerable merits of monolithic homogeneous integrated MSCs in high power output, their intrinsic drawbacks are likewise magnified at the array dimension and constitute key impediments to practical system deployment. First, from the perspective of energy storage mechanisms, constrained by the predominantly electric double layer capacitive charge storage and the planar architecture, their areal and volumetric energy densities remain relatively low, rendering them inadequate for sustained power supply or high energy density applications [40]. Second, a more critical issue lies in the amplification effect of cell‐to‐cell variation. In series‐connected MSC arrays, minor deviations in ESR or capacitance among individual cells can induce uneven voltage distribution, thereby constraining the overall output voltage and compromising system reliability [16]. In high‐frequency applications, such parametric dispersion additionally introduces extra phase lag and energy dissipation. Furthermore, the thermal budget constraints imposed by Complementary Metal‐Oxide‐Semiconductor (CMOS) back‐end‐of‐line (BEOL) integration constitute a critical bottleneck for monolithic MSCs fabrication. Standard CMOS BEOL processes typically restrict post‐processing temperatures to below 400°C–450°C to preserve the integrity of interlayer dielectrics, Cu interconnects, and low‐κ materials. However, several high‐performance MSCs electrode materials require synthesis or activation temperatures that substantially exceed this threshold. For instance, CDC formation via TiC chlorination demands temperatures of 600°C–900°C, while crystalline CNT growth via chemical vapor deposition typically requires 600°C–750°C. Even solution‐processed MXenes often necessitate thermal annealing at 300°C–500°C to achieve optimal electrical conductivity and surface termination control, approaching the upper limit of BEOL compatibility. Table 1 summarizes these process windows, revealing that only a limited subset of material systems, including room‐temperature electrodeposited PEDOT:PSS/graphene composites and low‐temperature inkjet‐printed nAC, fully satisfy CMOS thermal constraints without performance trade‐offs. This mismatch in temperature values prevents the seamless monolithic integration of certain high‐performance materials with integrated circuits [24].

Building on the above analysis, monolithic homogeneous integration is particularly well suited for applications that are sensitive to parasitic effects or depend on the cooperative response of arrays, including on‐chip high‐frequency filtering, high‐voltage pulse energy shaping, and microsystems that demand stable voltage output [42, 89]. For such applications, minimizing energy transport pathways and enhancing structural uniformity are of greater importance than single‐device performance [16]. Nevertheless, this approach faces limitations including constrained material choices, high fabrication complexity, and inherent challenges in achieving higher energy density. Accordingly, the design of such systems should adhere to the following fundamental principle: prioritize fabrication approaches that offer high patterning resolution and high uniformity, and while maintaining process compatibility, suppress cell‐to‐cell variation through structural optimization and array design, so as to achieve stable system‐level performance output [76].

### 3.2 Monolithic Heterogeneous Integration

Monolithic heterogeneous integration involves the in situ integration of MSCs with other functional modules, including energy harvesting, sensing, or signal processing units on a single substrate [39, 103], enabling deep system‐level coupling of multiple functionalities via joint structures and fabrication processes. Unlike homogeneous integration, which prioritizes structural consistency, heterogeneous integration faces the central challenge of achieving interface compatibility and synergistic mechanisms among functionally distinct units [91]. Within these systems, MSCs function not merely as standalone energy storage components but rather take on system‐level roles including energy buffering, power conditioning, and signal stabilization, their performance must therefore be dynamically coordinated with that of other functional modules [48]. At the system level, the essence of monolithic heterogeneous integration is not the mere superposition of multiple functionalities, but rather the realization of efficient coupling among distinct physical processes. This coupling depends on three main factors: first, the material‐level properties of electrical conductivity and electrochemical activity must concurrently meet the demands of energy storage and other functional roles [91]. Second, the interfacial architecture should guarantee uninterrupted charge and ion transport, preventing additional resistance or hysteresis arising from interface incompatibility [48]. Third, the operating voltage, current, and temporal response characteristics across disparate functional units must be matched at the system level, failure to do so would result in reduced energy efficiency or signal distortion [104]. Hence, whereas homogeneous integration prioritizes parameter uniformity, heterogeneous integration underscores the importance of parameter matching and the efficiency of interfacial coupling.

Subject to the requirements of interfacial compatibility and parametric synergy, the design of materials establishes, at the source, the degree of complexity associated with heterogeneous integration. To mitigate the uncertainties arising from multi‐material interfaces, the recently proposed “single‐material‐platform heterogeneous integration” strategy achieves multifunctional differentiation within a single material platform by constructing carbon‐based hybrid material systems (e.g., carbon/metal oxide or carbon/conductive polymer) [91], thereby accommodating both energy storage and functional response requirements at the materials level [48, 104]. For instance, carbon materials supply a highly conductive network and structural scaffolding, whereas metal oxides or conductive polymers introduce functionalities such as pseudocapacitance or sensing response, thereby endowing a single electrode system with simultaneous capabilities for energy storage and functional output [48, 104]. Such hybrid designs involving “carbon and functional component” circumvent, to a certain extent, the issues of contact resistance and interfacial mismatch inherent in conventional heterogeneous interfaces, thereby benefiting the continuity of charge transport and system stability.

**FIGURE 4:**
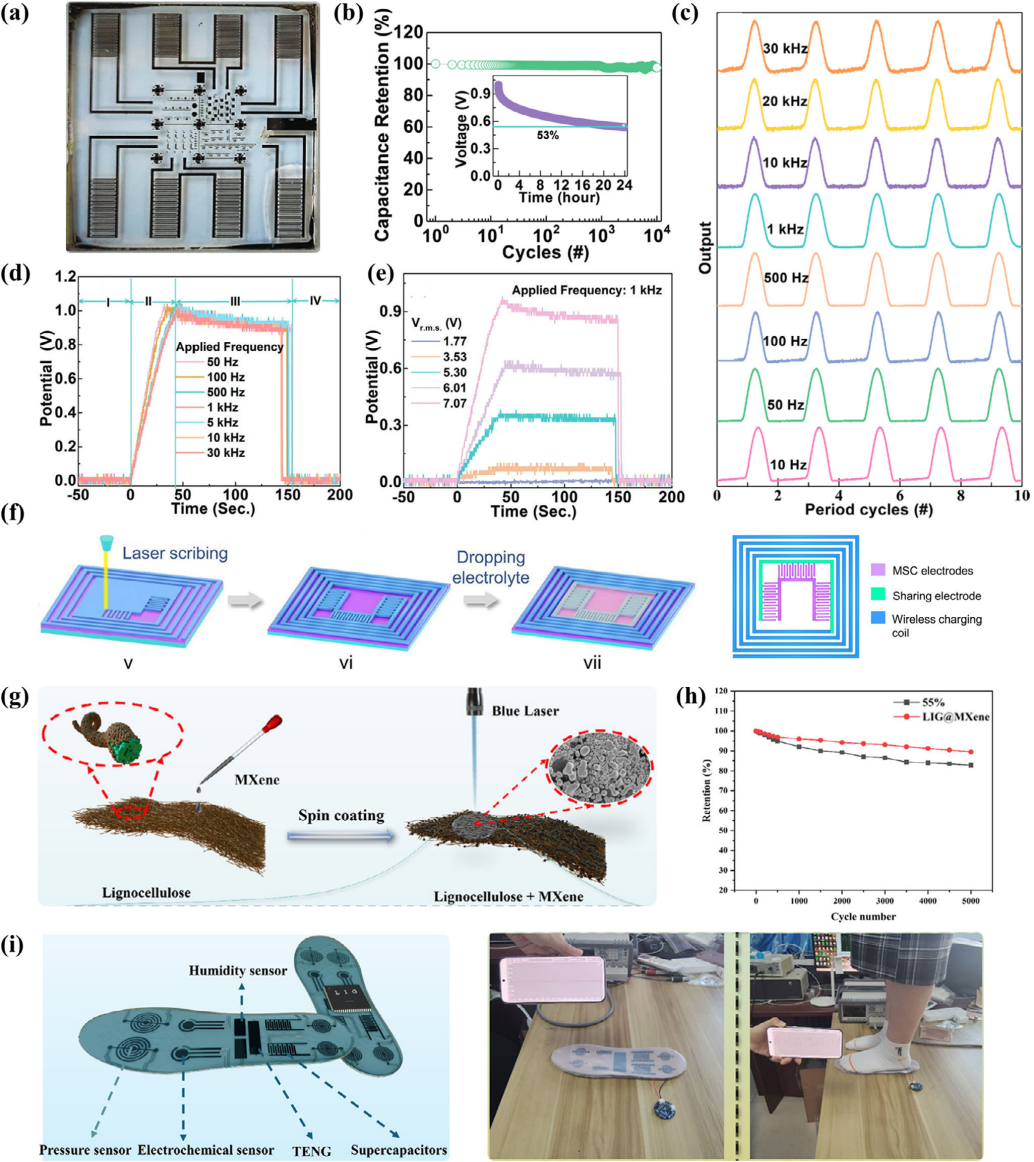
Fabrication and application studies of monolithically integrated heterogeneous MSCs. (a) MSCs‐thin film rectifier circuit integrated on a glass substrate. (b) Cycling stability and self‐discharge curves of the MSCs. (c) Half‐wave output signals obtained under input signals with different frequencies. (d) MSCs are charged with alternating current signals at various frequencies. (e) MSCs are charged using input signals of varying peak voltage magnitudes. Reproduced with permission from Ref. [92], Copyright 2019, Wiley‐VCH. (f) Fabrication process of the IWC‐MSC. Reproduced with permission from Ref. [91], Copyright 2024, The Author(s). (g) Preparation method of the LIG@MXene multifunctional composite material. (h) Capacitance retention of MSCs after 5000 cycles. (i) Self‐powered smart insole. Reproduced with permission from Ref. [104], Copyright 2025, The Author(s).

Beyond the materials level, the fabrication strategy further determines whether these functional units can achieve spatially controlled integration and effective coupling. Current monolithic heterogeneous integration primarily relies on microfabrication techniques such as patterning [91], thin film deposition [92], and lithography‐assisted integration to achieve precise definition and interconnection of distinct functional regions on a common substrate. First, micro‐nano processing techniques including photolithography and magnetron sputtering enable functional coupling at the device level. As shown in Figure 4a, Hota et al. [92] employed a RuO_2_‐based material co‐utilization strategy to monolithically integrate a rectifier and MSCs on a single substrate. By exploiting the conductivity and pseudocapacitive characteristics of RuO_2_, they realized the dual functionality of electrodes and gates within a shared architecture. The MSCs exhibited a self‐discharge rate as low as 18.75 mV·h^−1^, while the rectifier demonstrated a half‐wave rectification bandwidth spanning from 10 Hz to 30 kHz (Figure 4b,c). Under an alternating current signal input, the system realizes stable rectification and charge storage, thus fulfilling an on‐chip closed loop of “AC input to DC output” (Figure 4d,e). These findings suggest that material co‐utilization and interfacial simplification are effective strategies for enhancing the integration density of energy management systems. Second, laser direct writing has emerged as a key technique for monolithic heterogeneous integration owing to its spatially controllable energy input and material transformation capabilities. By employing selective reduction, etching, or phase transformation, this approach enables the creation of regions with distinct structures and functionalities within a single precursor material, thus facilitating the monolithic integration of multifunctional units. As shown in Figure 4f, Gao et al. [91] employed laser patterning to simultaneously fabricate energy storage electrodes and wireless charging coils from the PEDOT:PSS/[EMIM][TFSI] system, realizing an IWC‐MSC that integrates both energy harvesting and storage capabilities. MSCs can function as power buffering components within energy harvesting and storage systems. Nevertheless, such homologous heterointegration designs rely on a single material to fulfill multiple functions, introducing inherent trade‐offs among electrical conductivity, electrochemical activity, and electromagnetic response, thereby constraining further performance enhancement of the system. Additionally, multifunctional composite materials offer a route toward higher‐order functional integration. As shown in Figure 4g, Zhao et al. [104] fabricated an LIG@MXene composite architecture from a lignocellulose/MXene precursor using laser direct writing, they achieved monolithic integration of MSCs (exhibiting approximately 89.5% capacity retention after 5000 cycles, Figure 4h) with various sensing and heating modules on the same flexible substrate (Figure 4i), demonstrating the viability of self‐powered multi‐parameter monitoring systems. While this strategy substantially broadens system functionality, the coupling of charge and thermal transport at interfaces may introduce parasitic losses because of the involvement of multiple physical processes including electrochemical reactions, thermal dynamics, and resistive effects, which can compromise system stability and long‐term reliability.

With functional heterogeneity and interfacial matching accomplished, monolithic heterogeneous integration exhibits system‐level performance merits that surpass those of homogeneous integration, predominantly embodied in improvements in energy density and the realization of multifunctional cooperation. First, the incorporation of pseudocapacitive components (such as metal oxides) or conductive polymers into carbon‐based conductive scaffolds to form hybrid electrodes significantly augments the areal or volumetric energy storage capability, thus breaking through the energy density bottleneck that constrains traditional planar MSCs [48, 104]. Second, the tight monolithic integration of distinct functional modules (such as energy harvesters, sensors, and signal processors) converts MSCs from passive charge reservoirs into system‐level functional hubs, allowing them to collaboratively contribute to energy buffering, power conditioning, and signal fidelity maintenance [91]. This multifunctional interplay not only elevates the level of system integration but also diminishes the need for off‐chip wiring and associated energy losses, thus furnishing a pivotal foundation for the realization of exceptionally compact on‐chip microsystem architectures.

Although monolithically heterogeneously integrated MSCs exhibit distinct advantages in multifunctional coupling, their performance optimization confronts an intrinsic trade‐off between materials selection and interfacial compatibility. To reduce interfacial complexity, the currently prevalent “isogenous heterostructure” strategy relies on a single material system to simultaneously accommodate energy storage and other functions (such as sensing or rectification) [92]. However, constrained by the intrinsic properties of the material, such approaches rarely enable each functional unit to achieve its optimal performance, typically resulting in compromises among electrical conductivity, electrochemical activity, and functional responsiveness. Conversely, introducing distinct materials to individually optimize each functional module, while beneficial for enhancing unit performance, inevitably introduces heterogeneous interfaces, thereby giving rise to issues of interfacial mismatch [76]. Specifically, the discontinuity of conductive networks between different materials increases interfacial resistance, thereby diminishing the efficiency of energy and charge transport between the MSCs and other functional units. Concurrently, the presence of unstable contacts at these junctions may further jeopardize the cycling endurance and extended operational reliability of the integrated device. Therefore, for monolithically heterogeneously integrated MSCs, the central challenge is not the enhancement of any single performance metric, but rather how to strike a balance between “material diversity” and “interface controllability” [48, 104]. In essence, it entails attaining peak performance for each integrated function while concurrently suppressing parasitic losses originating from interfaces and augmenting the cross‐functional coupling efficiency.

Based on the above analysis, monolithic heterogeneous integration is more suitable for application scenarios that require functional synergy and system‐level integration, such as skin‐adherent electronic devices and systems on chip (SoC) [48, 92, 104]. Within such systems, MSCs serve as the interface between energy harvesters and loads, enabling energy buffering and management, which ensures reliable system operation under intermittent or low‐power conditions [48]. The design of such systems should follow these fundamental principles: prioritize material platforms that offer multifunctional capabilities or superior interfacial compatibility, optimize interfacial transport pathways via architectural engineering, and ensure system‐level matching of operating parameters including voltage, current, and response time across functional units, thereby achieving efficient and robust functional coupling.

## 4 Hybrid Integration

Hybrid integration is defined as the assembly of multiple discrete chips or modules into a system‐level unit using advanced packaging techniques, wherein a common substrate is not a prerequisite [105]. Within the system‐oriented framework proposed, hybrid integration corresponds to the coupling mode in which spatial proximity and packaging‐level interconnection are used to satisfy system constraints that cannot be fully addressed through monolithic co‐fabrication, such as material/process compatibility, functional scalability, and maintainability. Hybrid integration is mainly classified into two distinct forms: hybrid co‐packaged integration and modular hybrid integration [106, 107]. Analogous to System‐in‐Package (SiP) technology, hybrid co‐packaged integration encapsulates heterogeneous chips, including MSCs, sensors, and processors, within the same carrier or package body through stacking or lateral arrangement, thereby realizing tightly coupled, system‐level operation [108]. Under this approach, each functional chip may utilize its most favorable materials and fabrication processes independently, while high‐density interconnections (such as chip‐scale interconnects or wire bonding) ensure tight collaboration at the packaging level, thereby effectively reducing interconnect length and improving signal and power delivery efficiency [105, 108]. Conversely, modular hybrid integration prioritizes the physical and functional disaggregation of functional blocks. Within this framework, MSCs operate as autonomous energy storage units that connect to other modules through external wiring or standardized interfaces [109, 110]. While this strategy incurs penalties in volumetric efficiency and transient response characteristics, it substantially bolsters system scalability and reconfigurability, facilitating expedited upgrades in alignment with evolving demands. Accordingly, this section focuses on how packaging‐level coupling and functional decoupling can be selected according to system constraints, thereby extending the proposed integration logic from monolithic co‐fabrication to more flexible microsystem assembly pathways.

### 4.1 Hybrid Co‐Packaging Integration

When system design requires a balance between energy transfer efficiency and process flexibility, hybrid co‐packaged integration offers an intermediate paradigm between monolithic integration and module‐level integration [32, 35]. In contrast to monolithic integration, which relies on intrinsic coupling through structure or interface, this strategy achieves spatial proximity and electrical interconnection of functional units at the packaging level, thereby constructing a tightly coupled microsystem. Within this framework, individual functional modules remain relatively independent physically, yet operate cooperatively at the system level via the packaging architecture, consequently, system performance is no longer governed solely by single device attributes but is jointly determined by the intrinsic properties of the components and the packaging‐interconnect configuration [32, 35]. This imposes explicit requirements on the interconnect pathways and packaging architecture, with the central objective being the reduction of interconnect resistance and the realization of high‐density, compact packaging. First, reducing the physical separation among functional components mitigates parasitic resistance and inductance, thereby improving the efficiency of both energy and signal transfer and facilitating faster transient response [111]. Second, the communal packaging medium (such as elastomeric polymers or potting compounds) serves dual roles in mechanical reinforcement and environmental shielding while simultaneously governing interfacial contact integrity and heat spreading characteristics [106]. Therefore, in contrast to monolithic integration where material homogeneity is paramount, hybrid integration prioritizes “spatial coupling efficiency,” namely the capability to realize low‐loss interconnects and effective thermal regulation within a limited volumetric envelope. As the integration density increases, the packaging must simultaneously satisfy multiple constraints including electrical insulation, mechanical flexibility, and long‐term reliability, rendering it a critical limiting factor in system design.

**FIGURE 5:**
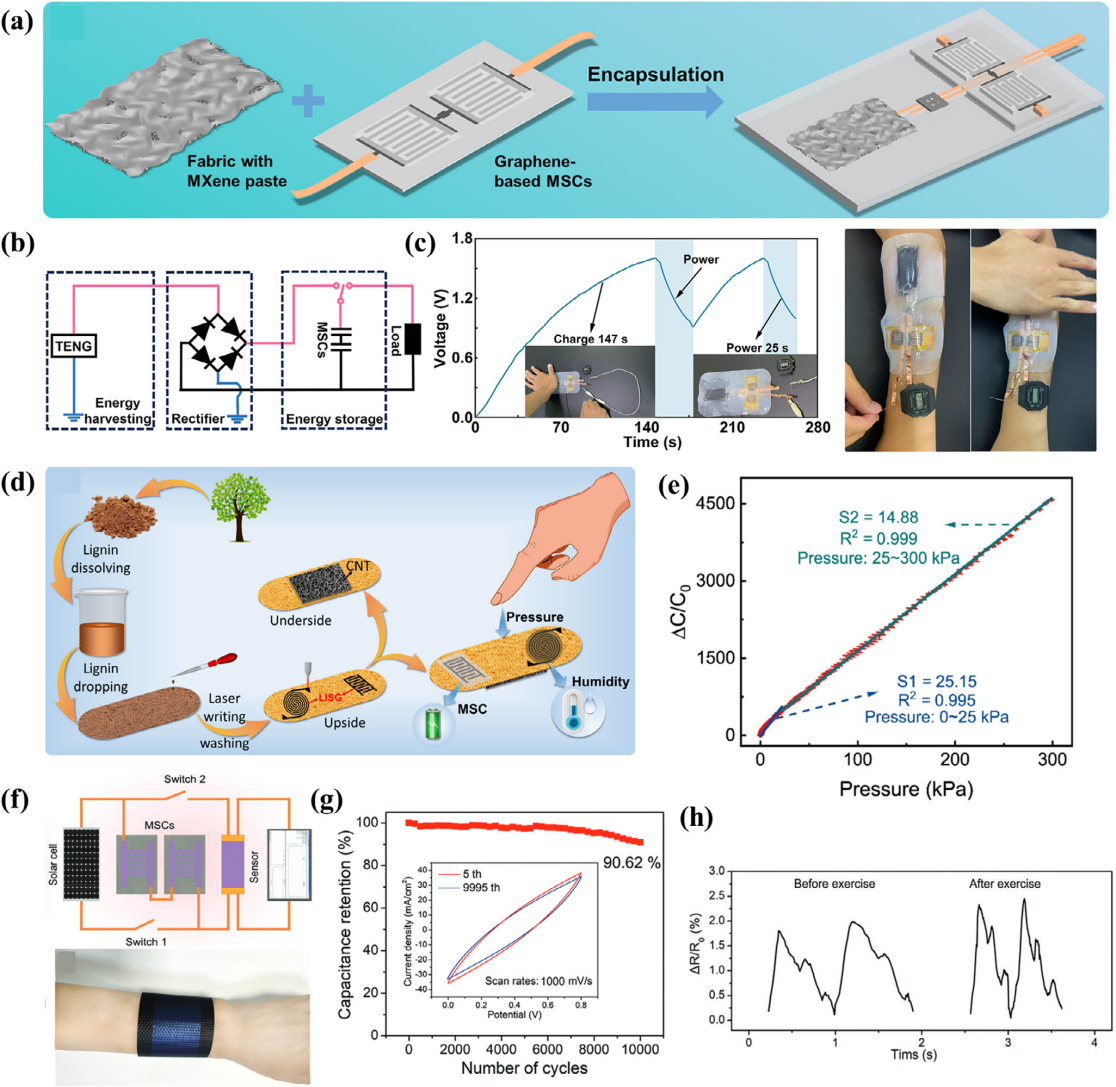
Fabrication and application studies of hybrid integrated MSCs. (a) A self‐powered system based on MXene and graphene. (b) Working principle of the self‐powered system. (c) Charge–discharge curves of the MSCs and a schematic diagram of powering an electronic watch. Reproduced with permission from Ref. [106], Copyright 2025, Central South University. (d) A self‐powered sensing system integrated on a bandage. (e) capacitance variation with applied pressure in the range of 0–300 kPa. Reproduced with permission from Ref. [37], Copyright 2022, Elsevier. (f) Wearable sensing device based on a flexible SEBS substrate. (g) Capacity retention of the MSCs after 10 000 cycles. (h) Real‐time monitoring curves of human pulse pre‐ and post‐exercise. Reproduced with permission from Ref. [41], Copyright 2023, Wiley‐VCH.

In specific applications, as shown in Figure 5a, Fan et al. [106] integrated an MXene‐based triboelectric nanogenerator (TENG) with graphene interdigitated MSCs within the same silicone rubber encapsulation layer, allowing the encapsulation to simultaneously serve as both the triboelectric interface and structural support, thereby achieving compact coupling of energy harvesting and storage units, and enabling the series‐connected MSCs to be charged to approximately 1.6 V under short‐term mechanical stimulation to drive low‐power devices (Figure 5b,c). This result demonstrates that shortening the interconnection path can significantly enhance transient power response, however, the packaging material must simultaneously satisfy both mechanical and electrical performance requirements, and the trade‐off between its elasticity and dielectric properties may affect long‐term stability. Furthermore, the co‐packaging strategy based on the same material system can reduce interfacial complexity. As shown in Figure 5d, Yuan et al. [37] employed laser‐induced sulfur‐doped porous graphene to monolithically integrate MSCs with humidity and pressure sensing components on a flexible substrate, enabling sensitive pressure detection via the minimization of heterogeneous interfaces and interconnection routes (Figure 5e). However, although this “quasi‐homogeneous strategy” is beneficial for reducing interfacial losses, it also faces the issue of limited functional coupling, namely that a single material system cannot simultaneously achieve optimal performance in terms of electrochemical properties and sensing sensitivity.

The core advantage of hybrid co‐packaged integration lies in its ability to realize independent optimization of individual functional units while maintaining system compactness. Since different devices can separately adopt optimal material systems and fabrication processes, their electrochemical performance, energy conversion efficiency, or sensing sensitivity can all attain high levels at the device stage, free from the constraints of a single material platform [106]. Concurrently, the spatial coupling afforded by the packaging layer enables these high‐performance units to operate cooperatively with low losses, thereby reconciling performance with integration density at the system level [105, 106].

However, compared with monolithic integration, the hybrid co‐packaged strategy still presents non‐negligible limitations, which primarily stem from parasitic losses and additional spatial occupancy. First, although interconnect traces may be considerably abbreviated, the inter‐device electrical linkages unavoidably incorporate parasitic resistance, leading to energy losses and propagation delays under high‐frequency operation or fast charging–discharging conditions [32]. Second, owing to the physical autonomy retained by each functional unit, the requisite layout margins and encapsulation perimeters consume supplementary area, thus impeding further dimensional downscaling of the overall system [35]. Moreover, the intrinsic thickness and dielectric attributes of the packaging layer can extend the effective path length for energy transport, progressively evolving into a limiting factor for performance in scenarios of elevated integration density [106]. Therefore, how to reduce parasitic effects while simultaneously shrinking the system footprint constitutes a critical issue for the further advancement of hybrid co‐packaged integration.

Based on the above analysis, hybrid co‐packaged integration is suitable for application scenarios that require a balance between performance and manufacturing complexity, demonstrating unique advantages particularly in wearable self‐powered systems and flexible electronic devices [37, 106]. In these systems, MSCs typically serve as energy buffering and power regulation units, connecting energy harvesting modules with functional loads to achieve efficient temporary energy storage and release. Their design should adhere to the following principles: minimizing interconnection path length through compact layout, selecting packaging materials that possess both mechanical and electrical stability, and optimizing the spatial distribution and operational matching of functional units at the system level, thereby enhancing overall coupling efficiency while maintaining flexibility [37, 106].

### 4.2 Modular Hybrid Integration

When application requirements prioritize capacity expansion, functional upgrades, and long‐term system evolution, the integration approach emphasizes functional decoupling and reconfigurability rather than extreme spatial compression. In modular hybrid integration, MSCs, as independent energy storage units, and functional devices remain relatively separate during the design and manufacturing stages [109, 110]. This integration approach results in a system with a simple structure, low manufacturing and maintenance costs, and is suitable for scenarios where space constraints are not critical and design flexibility is valued.

In terms of specific implementation, wearable self‐powered systems represent an important application scenario for modular integration. As shown in Figure 5f, Wang et al. [41] constructed a self‐powered sensing platform based on a SEBS flexible substrate, in which MSCs served as an independent energy module connected to sensing units via wires, achieving decoupling of energy supply and signal acquisition, the device exhibited a capacity retention of approximately 90.6% after 10 000 cycles and could stably support physiological signal monitoring (Figure 5g,h). This indicates that the modular structure ensures long‐term stability of the energy storage unit without compromising the functional output of the system. This strategy significantly reduces manufacturing complexity and allows for the replacement of individual modules in the event of system degradation or functional upgrades, thereby enhancing the overall maintainability and service life of the system.

From a system‐level perspective, the core advantage of modular integration lies in decomposing complex systems into relatively independent functional units, thereby reducing design coupling and enhancing system robustness [109, 110]. First, the decoupling between energy storage modules and functional modules allows each unit to be designed and optimized under its own optimal conditions; for example, MSCs can independently improve cycling stability and capacity, while sensing modules can prioritize optimization of sensitivity and response time. Second, standardized electrical connections between modules enable energy and signal transmission, endowing the system with flexible replaceability and upgradeability [109, 110]. Finally, during system expansion, the number of MSC modules can be increased through series or parallel configurations to achieve adjustable scaling of output voltage or capacity [107]. Therefore, in contrast to monolithic and co‐packaged integration, which emphasize “coupling efficiency,” the modular strategy focuses more on system‐level evolvability and configuration flexibility.

However, the cost of functional decoupling lies in the reduction of system coupling efficiency. As modules rely on external interconnections, parasitic resistance and contact resistance are inevitably introduced during energy and signal transmission, leading to energy loss and response delay. Furthermore, longer transmission paths further amplify these parasitic effects under high‐frequency or pulsed operating conditions, limiting the system's transient power output capability. At the same time, modular design typically requires additional packaging and interface structures, which to some extent increases system volume and reduces spatial utilization efficiency [109, 110]. Therefore, modular integration exhibits a classic trade‐off between “flexibility and performance.”

Based on the above analysis, modular hybrid integration is more suitable for application scenarios with high requirements for system flexibility and scalability, such as wearable health monitoring platforms, reconfigurable sensor networks, and long‐term deployed self‐powered systems [41]. In these systems, MSCs typically serve as independent energy modules, adjusting output characteristics through series and parallel configurations, and combining with different functional modules on demand to achieve system‐level functional expansion. Their design should adhere to the following principles: prioritizing the reduction of parasitic parameters in inter‐module interconnection paths, enhancing module compatibility through standardized interfaces, and balancing energy transmission efficiency with structural flexibility at the system level, thereby achieving synergistic optimization of performance and scalability [109, 110].

### 4.3 Selection Logic for Integration Approaches

Within a system‐oriented design framework, the selection of an integration approach for MSCs is no longer a matter of a single manufacturing path but rather a design decision process that maps system requirements onto structural architecture (architecture mapping). In essence, different integration strategies correspond to distinct choices of “coupling modality,” representing a trade‐off among intrinsic coupling (monolithic), spatial coupling (co‐packaged), and functional decoupling (modular). Therefore, the logic governing this selection can be summarized into the following interrelated core dimensions.

#### 4.3.1 Coupling Efficiency vs. Footprint

When the system is highly sensitive to parasitic effects or requires extremely short energy transmission paths and stable high‐frequency or high‐voltage output, monolithic integration should be given priority [112]. This strategy achieves the lowest parasitic parameters and the highest energy transmission efficiency by eliminating or minimizing interconnect paths [16, 101]. In comparison, co‐packaged integration achieves a trade‐off optimization through spatial proximity [106], whereas modular integration, relying on external interconnections, exhibits the lowest coupling efficiency but offers the greatest structural freedom [41]. Therefore, this dimension essentially reflects the trade‐off between “minimum loss” and “maximum flexibility.”

#### 4.3.2 Material/Process Compatibility

When there is incompatibility between the energy storage unit and functional modules in terms of process temperature, material systems, or chemical environments, monolithic integration faces limitations in thermal budget and interfacial stability, which may lead to performance degradation or reliability issues. In this context, hybrid integration (particularly the modular approach) allows MSCs and other devices to be fabricated under their respective optimal conditions, for instance, MSCs can have their electrochemical performance optimized under high‐temperature or special environments, while CMOS or flexible devices retain their process integrity [109, 110]. Additionally, post‐fabrication screening and assembly strategies also contribute to improving system yield. Hence, this dimension defines the trade‐off between achieving performance limits and ensuring manufacturing feasibility.

#### 4.3.3 System Complexity vs. Evolvability

For systems requiring multi‐functional coordination or later‐stage upgrades, modular integration offers the highest scalability and reconfigurability through functional decoupling, enabling the system to dynamically adjust its structure and functional configuration according to demand [109, 110]. In contrast, co‐packaged integration provides limited extensibility while maintaining a certain level of coupling efficiency, making it suitable for moderately complex systems. By comparison, monolithic integration, owing to its highly rigid structure, is more suitable for systems with clearly defined functions and long‐term stable operation [23, 101]. Hence, this dimension captures the balance between system stability and the capacity for evolution.

**FIGURE 6:**
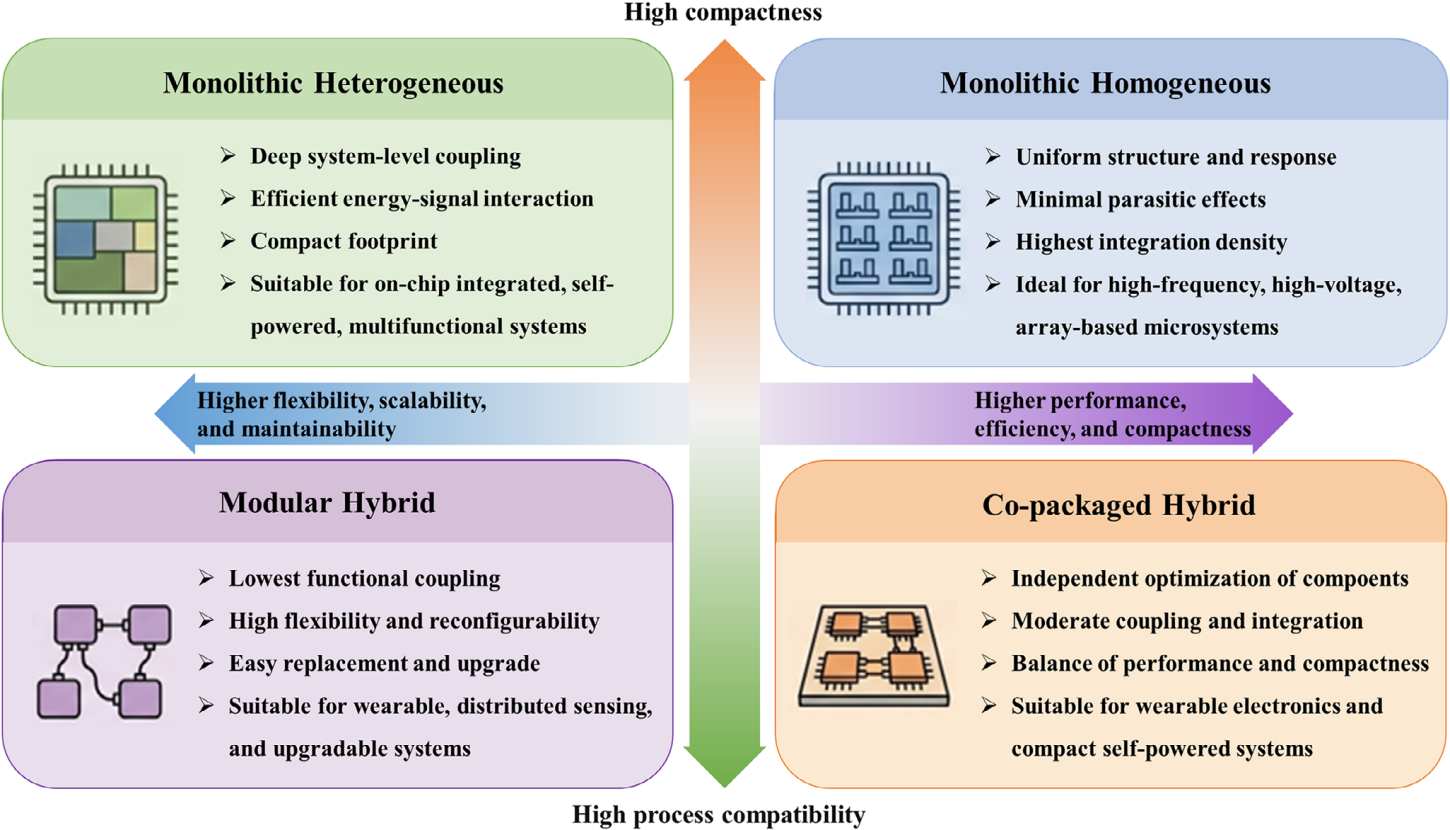
Integration selection phase map for MSC‐based microsystems.

Considering the factors discussed above, the choice of integration strategy for MSCs can be conceptualized as a continuous adjustment across three representative architectures. Monolithic integration is suited for high‐performance microsystems that demand maximal coupling efficiency and minimal area occupation, co‐packaged integration provides a trade‐off between performance and flexibility, modular integration caters to systems requiring scalability and reconfigurability. No single approach is universally superior, instead, each corresponds to the optimal solution under specific constraint conditions. Table 2 systematically compares integration strategies from various dimensions, providing a structured reference for design considerations. To further enable intuitive decision‐making, Figure 6 presents a phase map based on integration density and functional coupling, which visually summarizes the selection criteria and inherent trade‐offs among these strategies. Meanwhile, this selection logic also provides the basis for interpreting application‐dependent roles of MSCs in Section 5.

**TABLE 2:** Comparison between monolithic integration and hybrid integration.

Aspect	Monolithic integration	Hybrid integration
System design orientation	Compactness and consistency	Flexibility and scalability
Energy transmission path	Extremely short path, minimal parasitic effects from interconnects	Longer path, with additional interconnect losses
Material and structural freedom	Relatively low	Wide selection, highly customizable
Cmos compatibility	Poor	Good
Feature extension and refactoring	Limited	Easy to expand or refactor
System stability and consistency	High	Depends on packaging and interconnect reliability
Suitable application	On‐chip functional modules, high‐speed response stems	Multi‐functional systems, platforms with high energy demands, scalable systems

## 5 System‐Level Application and Functions of MSCs

This part concentrates on MSCs deployed in applications where they constitute an integral part of a fully functional microsystem. In these systems, MSCs are no longer isolated energy storage units; instead, they are embedded as intrinsic power modules or functional nodes within the microsystem, operating synergistically with energy harvesting, sensing, and signal processing units. In the system‐oriented framework developed, this part serves to verify how the proposed integration logic is translated into practical application scenarios, including wearable electronics [43], self‐sufficient sensor systems [41], pulsed energy scavenging [42], and AC line filtering [40]. Particular attention is given to how different integration routes shape the operational role of MSCs within complete microsystems.

### 5.1 MSCs as Power Buffers for Wearables

As flexible electronics and human‐machine interface technologies continue to evolve, wearable systems introduce multifaceted constraints on miniaturized energy storage units, encompassing mechanical conformability, instantaneous power responsiveness, and sustained operational reliability. Within these systems, MSCs generally transcend their function as mere energy storage components, instead undertaking system‐level functions including energy buffering, power regulation, and transient energy delivery, thereby addressing the intermittent energy harvesting and dynamic load requirements associated with human movement [5]. Consequently, the integration strategy for MSCs must achieve a coordinated optimization across mechanical compliance, power transfer efficiency, and functional integration intensity. Based on differences in application requirements, wearable systems can be broadly categorized into two typical scenarios. One targets continuous power supply, emphasizing system stability and scalable manufacturing [43], the other is oriented toward skin‐adherent electronics, highlighting ultra‐thin conformability, multi‐functional integration, and high interfacial reliability [48]. The two scenarios consequently place intrinsically distinct demands on the system role and integration strategy of MSCs.

#### 5.1.1 Energy Storage for Wearables

For wearable energy storage systems centered on sustained power delivery, MSCs mainly serve as stable energy delivery units and power buffering modules, designed to mitigate input energy fluctuations and enable the continuous operation of low‐power components. The critical requirement for this class of systems is to sustain stable electrochemical performance under dynamic mechanical deformation, such as stretching and bending, while simultaneously preserving scalability in manufacturing. Consequently, the integration approach for these systems typically leans toward monolithic homogeneous integration, where structurally consistent MSCs arrays are employed to minimize cell‐to‐cell variation, thus enabling reliable system‐level output [43].

**FIGURE 7:**
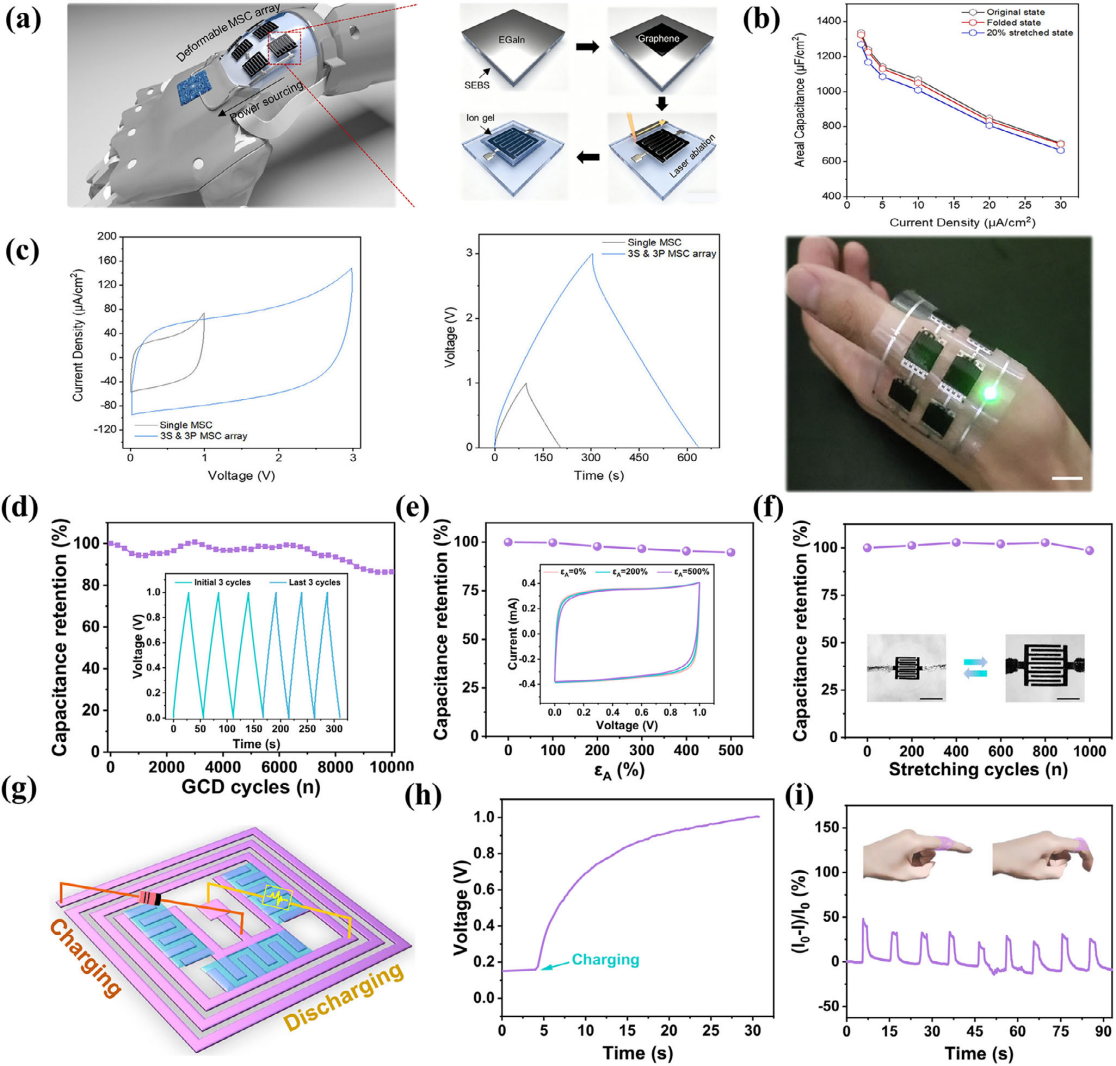
Integration and application of MSCs in wearables. (a) Schematic diagram of the integrated flexible MSCs system and the fabrication process of EGaIn‐based MSCs. (b) Rate capability of the flexible MSCs. (c) CV and GCD curves of series‐connected MSCs and a photograph of them powering an LED. Reproduced with permission from Ref. [113], Copyright 2024, The Author(s). (d) Cyclic stability of the MSCs. (e) Capacitance retention of the MSCs under varying levels of areal strain. (f) Capacitance retention over tensile cycling with areal strains reaching 500%. (g) Schematic diagram depicting the wireless charging and sensing operation of the microsystem. (h) Wireless charging profile of the MSCs subjected to 500% areal strain. (i) Current response of the strain sensor, powered by the WRC‐MSCs, under finger bending deformation. Reproduced with permission from Ref. [48], Copyright 2025, The Author(s).

For designs seeking to achieve greater mechanical compliance, the incorporation of deformable current collectors offers a viable strategy. As shown in Figure 7a, Woo Kim et al. [113] constructed monolithically integrated MSCs based on SEBS elastomer and eutectic gallium‐indium liquid metal, achieving simultaneous patterning of electrodes and interconnects via laser ablation. The resulting devices exhibited an areal capacitance of approximately 1336 µF cm^−^^2^ (Figure 7b) and maintained about 90% capacitance retention after repeated stretching or folding, while also being capable of stably driving LEDs through series and parallel connections (Figure 7c). These findings indicate that through optimization of current collector architecture and structural design, substantial improvements in mechanical robustness and reliable power delivery can be achieved without compromising the uniformity inherent to monolithic homogeneous integration. However, it should be noted that liquid metal electrodes may undergo irreversible flow‑induced agglomeration or surface oxidation after thousands of bending or tensile deformation cycles, thereby disrupting the continuous conductive network of the electrode. In parallel, the mechanical interface between the graphene layer and the liquid‑metal current collector may degrade under cyclic stress, which results in elevated internal resistance and thus diminishes the ability for transient power delivery.

#### 5.1.2 Conformal Skin Electronics

In contrast to energy storage systems centered on power delivery, skin‐mountable electronics, such as electronic skin, introduce tighter requirements for the system, encompassing ultra‐thin conformability, multifunctional integration, and sustained interface reliability. Within these systems, MSCs transcend their role as mere energy delivery components, functioning instead as nodes for energy regulation and functional integration that connect sensing, communication, and energy harvesting subsystems. Consequently, the integration approach for these systems favors monolithic heterogeneous integration, enabling efficient coupling of diverse functional units within limited spatial footprints [114].

At the system level, this approach enables coordinated functionality across diverse modules by combining parameter matching with interface optimization, while circumventing the parasitic losses and interfacial reliability issues inherent to conventional interconnections. As shown in Figure 7d, Ren et al. [48] utilized a pre‐wrinkled elastomeric substrate to inhibit crack propagation in MXene films during substantial deformation and exploited its multifunctional characteristics to co‐integrate a wireless receiver coil, MSCs, and a strain sensor upon a unified substrate. Among these components, the MSCs demonstrated robust cycling endurance, with a capacitance retention of 86.4% following 10 000 GCD cycles (Figure 7e). Moreover, the MSCs maintained excellent capacitance retention and cycle life across a range of applied strains (Figure 7f,g). Further, the microsystem accomplished wireless charging in about 20 s while subjected to 500% strain (Figure 7h) and successfully realized finger movement detection (Figure 7i), thereby attesting to its high‐efficiency coupling performance in a monolithic energy harvesting‐storage‐sensing system. However, during repeated large‑amplitude stretching‑releasing cycles, deformation mismatch among the functional layers (MXene electrode, liquid metal current collector, and elastic encapsulation layer) may induce interfacial microcracks or delamination, which elevate contact resistance and accelerate electrolyte penetration, thereby causing the overall energy transfer efficiency of the system to degrade significantly with increasing mechanical cycling number.

### 5.2 MSCs as Energy Nodes in Self‐Powered Sensing

In self‐powered sensing systems, MSCs are no longer responsible for sustained power delivery, rather, they function as a power buffering and scheduling node that interfaces between energy harvesting units and functional loads. Their essential function is to reconcile the disparity between intermittent energy harvesting and continuous or event‐triggered energy consumption, thus ensuring reliable system operation. Consequently, in contrast to energy density, these systems prioritize the power responsiveness, ESR, and power matching characteristics of MSCs [83, 115]. From a systems viewpoint, self‐powered sensing systems may be conceptualized as a dynamic sequence encompassing energy harvesting, storage, and utilization, wherein the energy flow is characterized by distinctly transient behavior. MSCs establish a temporal buffer between energy input and output through rapid charge–discharge processes, thereby preventing sensor failure caused by power fluctuations. Based on differences in system complexity and coupling degree, such systems can be further categorized into three typical architectures: modular systems [107], co‐packaged/multifunctional systems [39], and highly integrated on‐chip systems [116], which correspond to distinct energy management strategies and integration approaches.

#### 5.2.1 Wearable Self‐Powered Sensing

In wearable self‐powered sensing scenarios, a clear distinction must first be made from the wearable energy storage systems discussed in Section 5.1. Although both belong to flexible wearable electronics in terms of physical form, their system operation paradigms exhibit fundamental differences: wearable energy storage systems typically achieve continuous power supply based on pre‐stored energy, with design goals of stable output and long‐term endurance [43, 113]. Whereas self‐powered sensing systems rely on intermittent energy input from the environment or the human body (such as mechanical motion or light), with system operation driven by real‐time energy flow, and their core lies in the dynamic matching among energy harvesting, storage, and utilization [117]. Hence, in the latter systems, MSCs function not as the main power supply, but instead as a transient energy buffer that bridges the energy source and the load.

**FIGURE 8:**
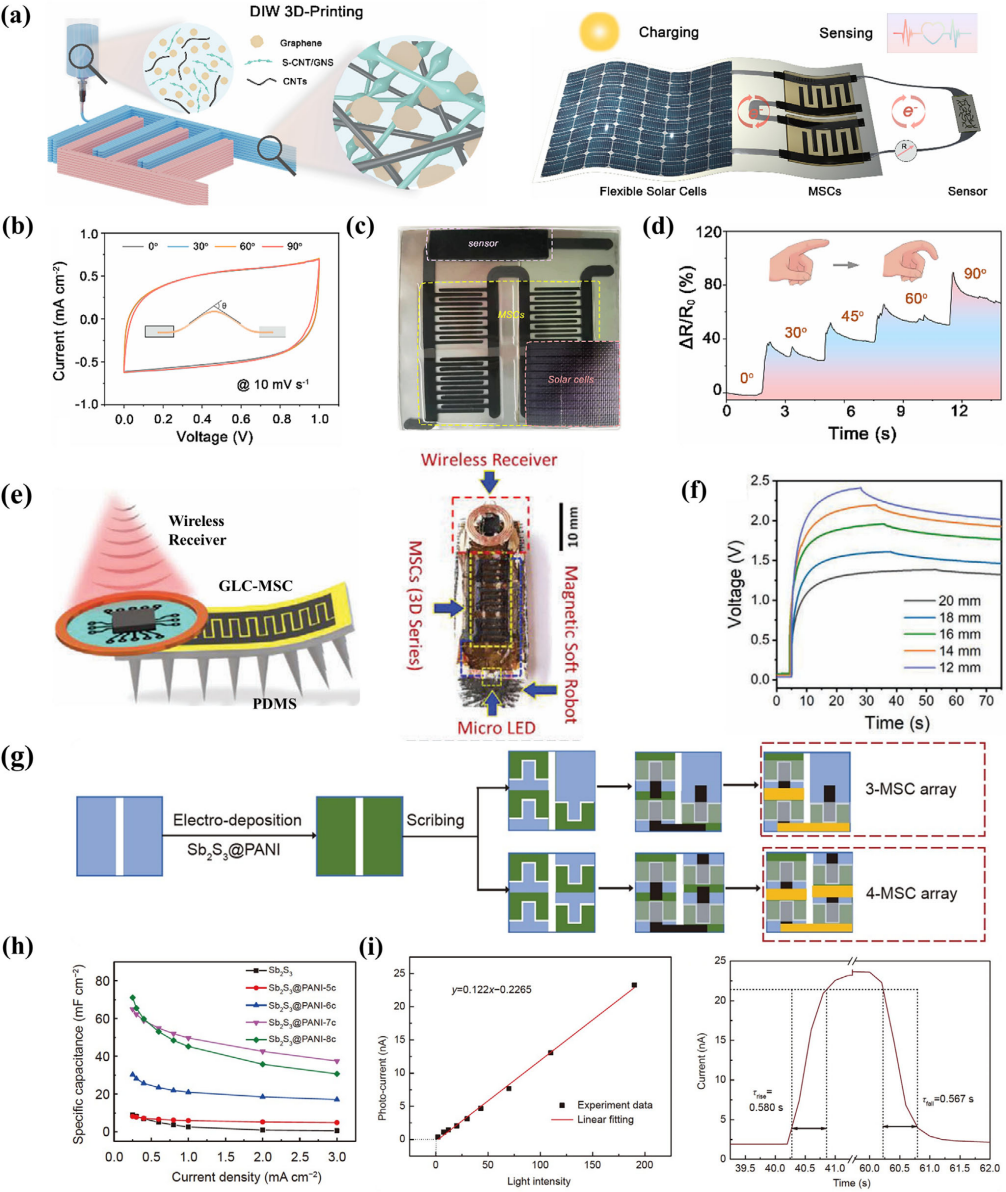
Integration and application of MSCs in self‐powered sensing systems. (a) 3D‐printed MSCs and a model of a wearable self‐powered sensing system. (b) CV curves of flexible MSCs under different bending angles. (c) Digital photograph of a flexible self‐powered system. (d) Finger motion records at different bending angles. Reproduced with permission from Ref. [107], Copyright 2024, American Chemical Society. (e) Soft robot equipped with GLC‐MSC and wireless charging system. (f) Wireless charging and self‐discharge curves of GLC‐MSCs. Reproduced with permission from Ref. [39], Copyright 2023, The Authors. (g) Schematic illustration of the preparation of in‐plane integrated AC//Sb_2_S_3_@PANI microelectrode arrays. (h) Rate capability of the MSCs, (i) photocurrent dependence on light intensity measured under the bias voltage provided by a 3‐MSC array. and the rise and decay response curves of the photodetector driven by the 3‐MSC array under a light intensity of 190 mW cm^−^^2^. Reproduced with permission from Ref. [116], Copyright 2024, Science China Press.

In these systems, the energy supply is characterized by considerable stochasticity and unpredictability, whereas sensing operations generally consist of low‐power, intermittent sampling events. Consequently, system design prioritizes low cost, ease of maintenance, and structural adaptability over the pursuit of ultimate energy transfer efficiency. Against this backdrop, MSCs function mainly to mitigate input fluctuations via fast charging and discharging, thereby delivering stable power to the sensing module [117]. Given these requirements, this class of systems generally adopts modular heterogeneous integration, enabling the independent optimization and interchangeable deployment of energy harvesting, storage, and sensing components. As shown in Figure 5d,f [37, 41], prior studies have already established the benefits of modular architectures in terms of lowered fabrication complexity and improved system expandability. Furthermore, Ren et al. [107] constructed a fully flexible self‐powered physiological monitoring system (Figure 8a), achieving an areal capacitance of approximately 0.47 F cm^−^^2^ for MSCs fabricated via 3D printing, with stable performance maintained under bending conditions (Figure 8b). A system comprising a solar cell, MSCs, and a stretchable sensor was integrated using flexible interconnects, enabling real‐time monitoring of finger movements (Figure 8c,d). These findings indicate that within loosely coupled architectures, MSCs can fulfill system demands solely through temporal energy buffering, obviating the necessity for intricate interfacial or structural integration.

#### 5.2.2 Multi‐Functional Self‐Powered Systems

In multifunctional self‑powered systems, the sensing, communication, and actuation components typically function concurrently, yet their power requirements differ substantially across both time and amplitude domains. This feature of asynchronous multi‐load operation places elevated requirements on MSCs, necessitating not merely energy storage but also the capability for dynamic power allocation and management [63, 64, 65].

For these systems, a completely modular architecture incurs considerable interconnection losses, whereas full monolithic integration faces limitations due to process and material incompatibilities. Hence, a co‐packaged approach is commonly employed to achieve improved coupling efficiency while preserving some level of flexibility. As shown in Figure 8e, Nardekar et al. [39] integrated MSCs, a wireless receiver, and PDMS into a single soft robotic system, enabling the coordinated operation of wireless energy delivery (Figure 8f) and sensing functionality. In this system, different functional units (such as deformation sensing and actuation response) have asynchronous energy demands. By means of rapid charging and discharging, MSCs modulate energy allocation across the time domain, thus ensuring sustained system operation and consistent responsiveness.

#### 5.2.3 On‐Chip Systems

In on‐chip sensing systems, the energy storage and sensing components are integrated onto a single substrate, giving rise to a tightly coupled microsystem. At this scale, the energy transport path is extremely short, but the system is highly sensitive to parasitic parameters and interfacial matching [118, 119, 120]. Therefore, such systems place greater emphasis on minimizing energy loss and improving response speed rather than on structural flexibility.

Given this demand, monolithic heterogeneous integration emerges as the dominant approach, leveraging materials and interfacial engineering to achieve coordinated integration of energy storage and sensing functionalities. As shown in Figure 8g, Chen et al. [116] constructed a PANI/Sb_2_S_3_ composite structure on a single substrate via electrochemical deposition, achieving the integration of MSCs and photodetection functionality. The device exhibited an areal capacitance of approximately 18.44 mF cm^−^^2^ (Figure 8h), and through a compact coplanar layout, significantly reduced energy transport losses, enabling the system to maintain stable response across a wide range of light intensities with a response time of approximately 0.58 s (Figure 8i). These findings indicate that monolithic integration, through the reduction of energy transport distances and the optimization of interfacial compatibility, enables fast energy conversion and superior response stability.

### 5.3 MSCs as Voltage‐Conditioning Units for Energy Harvesting

In pulsed energy harvesting systems, the input energy generally takes the form of transient signals that exhibit both temporal discontinuity and voltage irregularity, while the power density and peak voltage show substantial variation depending on the application scenario. This type of energy cannot be directly harnessed by microsystems, requiring instead a conditioning stage to render it usable [55]. In this process, MSCs no longer function merely as energy storage devices but instead assume the role of energy conditioning, converting unstable input into stable output capable of driving loads through energy buffering, voltage regulation, and power shaping [117]. From a system perspective, the usability of pulsed energy is primarily determined by three key parameters: pulse duration, peak voltage, and average power density. Different combinations of these parameters will directly determine the operating mode of MSCs and their integration strategy. Therefore, pulsed energy harvesting systems can be categorized into two typical architectures: low‐power discrete input systems and high‐voltage transient input systems [106], which correspond to distinct energy regulation mechanisms and structural design pathways.

#### 5.3.1 Low‐Power Ambient Energy Harvesting

In ambient energy harvesting (such as triboelectric, electromagnetic, or structural vibration), the input energy typically exhibits low average power density and random temporal distribution. Although such energy can be generated frequently, the energy per individual pulse is insufficient to directly power a load, therefore, the core of the system lies in achieving energy usability through temporal accumulation. In this process, MSCs primarily serve as energy buffering units, converting discrete input into quasi‐continuous output through continuous charge–discharge cycles [121, 122, 123].

Based on this mechanism, systems typically adopt hybrid co‐packaged integration to shorten the energy transport path and reduce losses during the buffering process. For example, in a self‐powered system driven by a TENG (Figure 5a–c), the power generation unit produces pulsed output with low voltage and low average power, while MSCs serve as an intermediate node to temporarily store the discrete energy. When the voltage reaches the operating threshold of the load, the MSCs rapidly release energy to drive the sensing or electronic devices [106]. This “accumulation‐trigger” mechanism effectively addresses the issue of low‐power input being unsuitable for direct utilization, validating the advantages of co‐packaged architectures in improving energy utilization efficiency.

#### 5.3.2 High‐Voltage Pulsed Energy Harvesting

In high‐voltage pulsed energy harvesting scenarios (such as droplet‐based electricity generation or electrostatic discharge), the input signal typically exhibits extremely high peak voltage and very short duration, yet the total energy is limited. The primary issue with such energy is not its insufficiency, but rather the mismatch between its voltage scale and the load, making it difficult to store or utilize directly [42]. Therefore, in such systems, MSCs primarily assume the functions of voltage matching and energy conditioning, namely reducing the voltage and enhancing charge availability while preserving the total energy.

**FIGURE 9:**
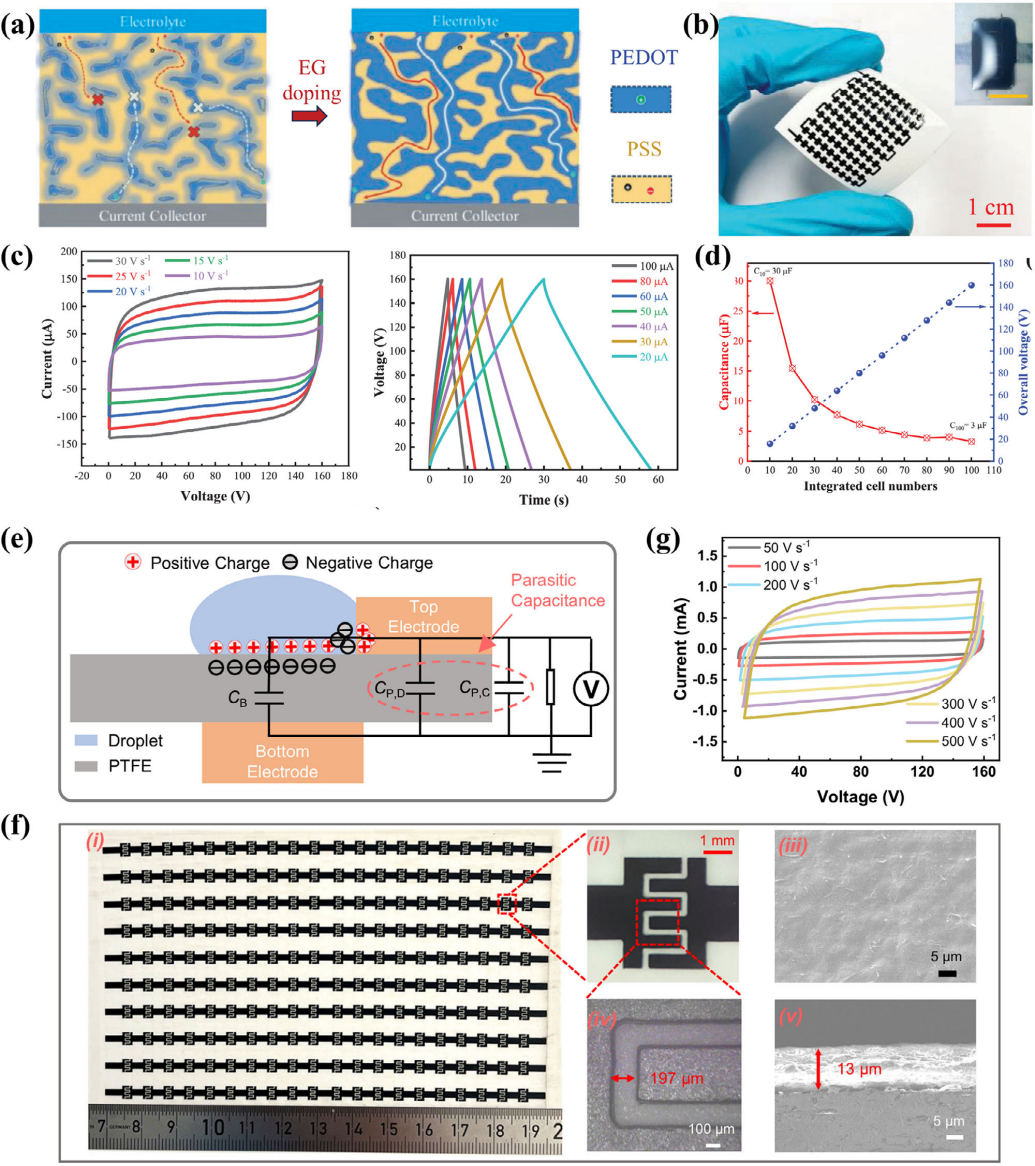
Integration and application of MSCs for high voltage pulsed energy harvesting. (a) Schematic diagram showing the doping‐induced phase separation of PEDOT‐rich and PSS‐rich domains. (b) 100 cells monolithically integrated in series over a footprint of 2.4 × 3.4 cm^2^. (c) CV and GCD curves of 100 series‐connected cells within a 160 V voltage window. (d) Total capacitance and operating voltage window plotted against the number of MSCs. Reproduced with permission from Ref. [42], Copyright 2024, The Authors. (e) Schematic representation of the localized bottom electrode (LBE) configuration along with its simplified equivalent circuit model. (f) A monolithic array comprising 400 integrated MSCs. (g) CV characteristics of the MSCs measured within a 160 V voltage window. Reproduced with permission from Ref. [102], Copyright 2025, The Author(s).

For efficient energy conversion, systems often adopt monolithically integrated high‐voltage MSCs arrays, where a series configuration improves the overall voltage withstand capability and minimizes parasitic losses from interconnects. As shown in Figure 9a, Li et al. [42] fabricated a monolithic MSCs array on a paper‐based substrate with EG‐doped PEDOT:PSS as the electrode material. By serially integrating 100 unit cells (Figure 9b), the system attained a voltage window of roughly 160 V and an equivalent capacitance of about 3 µF within a footprint of 2.4 × 3.4 cm^2^ (Figure 9c,d). Moreover, it could directly harvest a droplet‐generated electrical pulse of around 150 V, yielding an overall energy storage efficiency of 62%. In an optimized configuration depicted in Figure 9e [102], the incorporation of a localized electrode architecture boosted the output from the harvesting side, and the array was expanded to 400 cells, resulting in an exceptionally fast charging rate of 500 V s^−^^1^ (Figure 9f,g). The final system delivered an overall energy storage efficiency of about 21.8%, indicating that the combination of monolithic integration and voltage matching strategies substantially improves the utilization of high‐voltage pulsed energy. However, in a series array comprising hundreds of cells, the minor differences in capacitance or equivalent series resistance among individual units become accumulated and amplified during prolonged high‐voltage charge–discharge cycling, leading to uneven voltage distribution and overcharge or overdischarge of some units. For a large‑scale integrated system with 400 cells, the issue of heat dissipation under high‐density layout becomes even more prominent, in addition to cell uniformity. Repeated pulse charging and discharging at approximately 150 V generates a substantial amount of heat. If the heat cannot be effectively dissipated, it will cause uneven temperature distribution within the array, accelerate electrolyte decomposition and electrode aging, and ultimately lead to a significant decline in the overall energy storage efficiency.

### 5.4 MSCs as Frequency‐Selective Filtering Units

In circuit systems, suppressing mid‐to‐high frequency ripple is crucial for ensuring signal integrity and stable system operation [124, 125]. Traditional aluminum electrolytic capacitors, limited by their size, parasitic inductance, and high‐frequency response capabilities, can no longer meet the filtering requirements of on‐chip systems [126, 127]. MSCs, characterized by fast frequency response and low ESR, can effectively filter out ripple. In such systems, MSCs do not assume an energy storage role, instead, they are deployed as on‐chip filtering units near power supplies or loads to suppress signal noise. For effective filtering, system design critically depends on minimizing parasitic elements and reducing current path lengths. Hence, monolithic homogeneous integration is typically employed as the integration approach, utilizing MSCs arrays with uniform architecture and minimized interconnection distances to lower ESR, consequently improving high‐frequency responsiveness [89]. This approach is consistent with the earlier principle of monolithic integration, namely that structural uniformity minimizes parasitic effects, yet in this context it translates more explicitly into the optimization of frequency‐domain performance.

**FIGURE 10:**
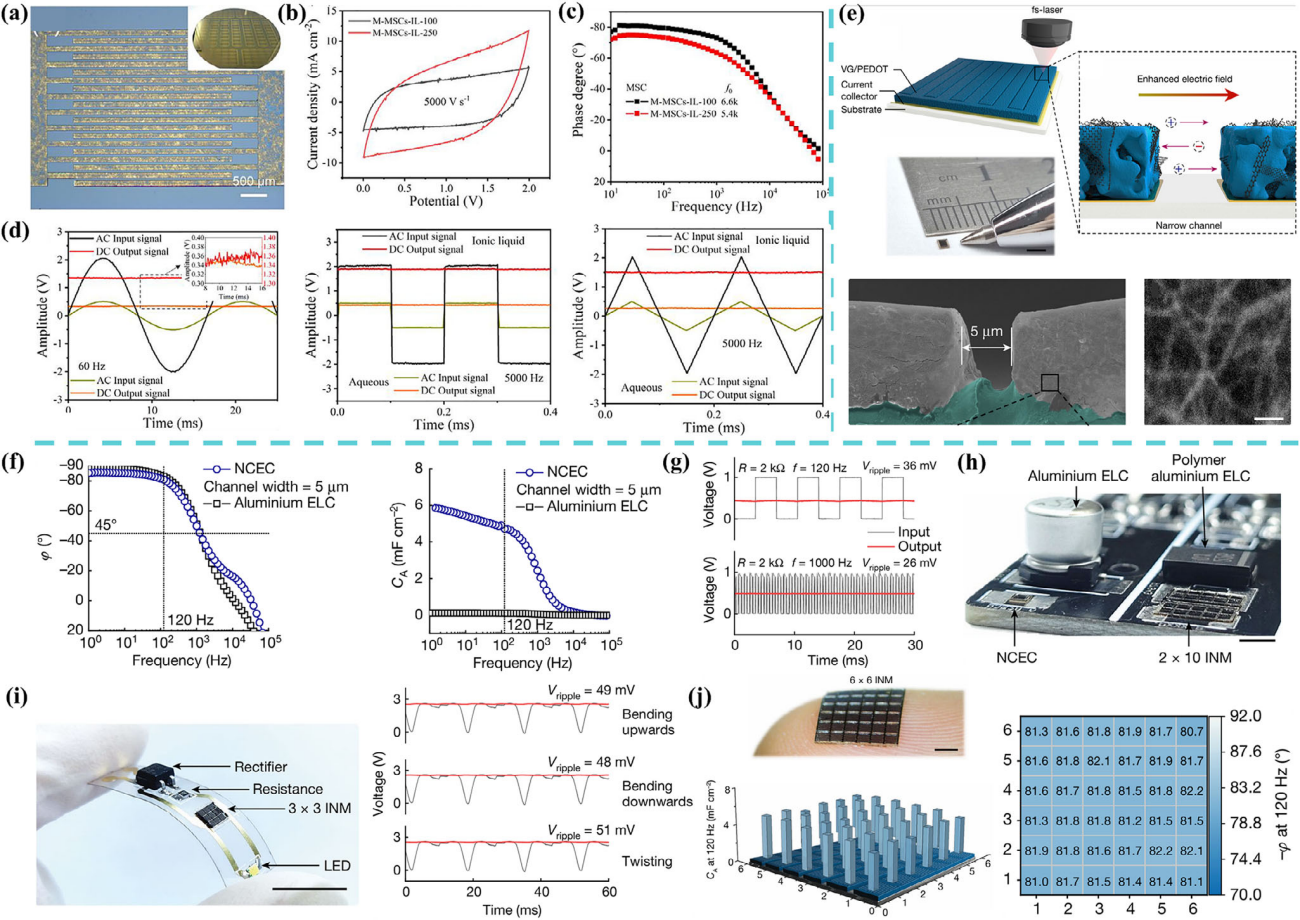
Integration and application of MSCs in circuit filtering. (a) MSCs integrated on a silicon chip. (b) Operating voltage and (c) characteristic frequency of MSCs in ionic liquid. (d) DC output signals after inputting 5000 Hz AC sine waves, square waves, and triangular waves. Reproduced with permission from Ref. [40], Copyright 2021, Science Press and Dalian Institute of Chemical Physics, Chinese Academy of Sciences. (e) Schematic illustration of femtosecond laser‐scribed electrodes alongside the 5 µm narrow‐channel electrode configuration. (f) Phase angle and areal capacitance of the narrow‐channel NCEC measured at 120 Hz. (g) Waveform diagrams of voltage signals after filtering by a single NCEC. (h) Comparison of the footprint volume between traditional aluminum electrolytic capacitors and the NCEC‐based integrated module delivering comparable performance. (i) Photograph of the flexible circuit along with voltage signal waveforms recorded under different mechanical deformation conditions. (j) Photograph of a 6 × 6 integrated NCEC array positioned on a fingertip, accompanied by the areal capacitance and phase angle values for each individual NCEC unit measured at 120 Hz. Reproduced with permission from Ref. [89], Copyright 2023, The Author(s).

As shown in Figure 10a, Feng et al. [40] developed monolithically integrated MSCs using two‐dimensional MXene for on‐chip AC line filtering. Owing to the high electrical conductivity of MXene and the broad operational voltage window afforded by the ionic liquid electrolyte (EMIMBF_4_), the device achieved an extended operating voltage of 2 V and exhibited a characteristic frequency of 6.6 kHz (Figure 10b,c). Under a high scan rate of 5000 V s^−^^1^, the MSCs efficiently converted a 5000 Hz AC input into a stable DC output (Figure 10d), indicating their sustained fast charge response capability even at elevated frequencies. These findings confirm that the mitigation of parasitic effects via monolithic integration substantially extends the effective frequency range over which MSCs can reliably operate. Despite the devices' outstanding filtering performance at the characteristic frequency of 6.6 kHz, under sustained high‑frequency operation, the parasitic reactions at the electrode/electrolyte interface (such as irreversible oxidation of surface functional groups on MXene) and parasitic inductance become amplified, leading to a gradual increase in equivalent series resistance and a deviation of the phase angle from its ideal value, thereby causing a slow degradation of the filtering performance over long‑term operation. Moreover, to increase the filtering frequency while minimizing system footprint, device architecture design emerges as a key enabling strategy. As shown in Figure 10e, Hu et al. [89] developed a narrow‐channel electrochemical capacitor (NCEC) featuring electrode channels of about 5 µm in width. This configuration leverages enhanced electric fields to expedite ion transport, consequently lowering the RC time constant and enhancing frequency response. At 120 Hz, the device demonstrated a phase angle near that of an ideal capacitor alongside exceptionally low resistance, and was capable of effectively attenuating ripple voltage (Figure 10f,g). The integrated module occupies roughly one‐tenth the volume of traditional aluminum electrolytic capacitors, yet retains stable filtering performance under flexing conditions, thereby achieving flicker‐free LED driving (Figure 10h,i). By synergistically optimizing structural scaling and monolithic integration (Figure 10j), this work enables a breakthrough in reconciling compact form factor with high‐frequency performance. The 6 × 6 array maintained stable performance after 200 000 cycles, representing a rare example of system‐level reliability validation. However, when maintaining a high‐density array, the electromagnetic crosstalk between cells may intensify with the aging process, thereby affecting the consistency of high‐frequency ripple suppression for the entire filtering module. This indicates that the reliability of array‐level filtering still requires more systematic evaluation.

### 5.5 Cross‐Scenario Summary of MSCs

In summary, the application of MSCs in microsystems has progressively evolved from standalone energy storage devices into multifunctional system units, covering diverse typical scenarios such as wearable electronics [48], self‐powered sensing [107], pulsed energy harvesting [42], and AC line filtering [89]. In this process, the functional role of MSCs has been significantly expanded, extending from conventional energy storage to critical system functions such as power buffering [48], energy regulation [39], voltage conditioning [42], and high‐frequency filtering [89], thereby enabling efficient coordination between various types of energy inputs and load demands.

From a systems viewpoint, these diverse applications can be collectively conceptualized as a matter of regulation across varying energy and signal scales. Specifically, in steady‐state power delivery scenarios, MSCs function mainly as continuous power sources [113]. Under intermittent energy input conditions, they provide energy smoothing through temporal buffering [107]. In pulsed energy systems, they enable voltage and power matching via energy conditioning [42]. In high‐frequency circuits, they exhibit the capability to filter AC signals [89]. Consequently, the role of MSCs in microsystems can be encapsulated as a continuous functional spectrum spanning energy storage, power regulation, energy conditioning, and high‐frequency filtering, where the particular form of implementation is dictated by the nature of the input energy and the system's operational frequency range.

Moreover, this evolution in functionality is intimately tied to the corresponding integration approach. Essentially, the requirements for coupling modes vary across different application scenarios, thereby dictating the structural realization pathways of MSCs. In on‐chip systems demanding high‐frequency or high‐power response, monolithic integration leverages structural uniformity and minimized energy transport distances to achieve low parasitic parameters and fast response, embodying the design principle of “structural consistency and parameter matching” [89]. In multifunctional collaborative or moderately complex systems, hybrid co‐packaging improves energy and signal transfer efficiency via spatial coupling [106]. In scenarios where system scalability and maintainability are paramount, modular integration enables system reconfiguration and upgrades through functional decoupling [39, 107]. These three integration strategies correspond respectively to intrinsic coupling, spatial coupling, and functional decoupling, forming a continuous design space ranging from high performance to high flexibility.

Hence, the integration of MSCs into microsystems is not to be viewed as a standalone functional realization, but rather as a process of orchestrating energy flow and signal flow under varying system constraints. The essence of their design resides in the selection of suitable integration approaches and device configurations based on input characteristics (steady‐state, intermittent, or pulsed), operational frequency (spanning from low to high), and system complexity, thus attaining an optimal trade‐off between energy efficiency, response speed, and system flexibility. Based on the above analysis, the diverse applications examined in Section 5 can be collectively integrated into a correlative scheme linking input characteristics, system requirements, functional role and integration strategy (presented in Table 3), thereby offering a comprehensive reference for the design and deployment of MEMS‐MSCs within microsystems.

**TABLE 3:** Comparison of MSCs across system‑level application scenarios: Input characteristics, system requirements, functional roles, and integration strategies.

Energy input characteristics	Application	System requirements	MSCs functional positioning	Integration method	Ref.
Steady‐state energy input	Wearable energy storage	Continuous power supply, structural consistency	Energy storage unit	Monolithic homogeneous	[113]
Multimodal biosignals, dynamic deformation	Electronic skin	Skin conformability, low interfacial impedance	Power buffering, functional synergy	Monolithic heterogeneous	[48, 91]
Intermittent energy input	Wearable sensing device	Power smoothing, low cost, maintainability	Power buffering, intermittent power supply	Modular hybrid/hybrid co‐packaging	[37, 41, 107]
AC or DC	Power management	Low parasitic parameters	On‐chip energy supply	Monolithic heterogeneous	[92, 116]
Multi‐source/multi‐load energy	Multi‐functional self‐powered system	Energy scheduling, functional synergy, system integration level	Energy scheduling node	modular hybrid	[39]
Low‐power pulse input	TENG‐driven system	Energy accumulation, triggered output, low loss	Power shaping	Hybrid co‐packaging	[106]
High‐voltage transient pulse	High‐voltage pulse energy harvesting	High voltage tolerance, low ESR, low leakage	Energy shaping, voltage matching	Monolithic homogeneous	[42, 102]
High‐frequency AC signal	On‐chip filtering, power	High‐frequency response, low ESR, high phase angle	Filtering	Monolithic homogeneous	[40, 89]

## 6 Summary and Outlook

### 6.1 Summary

Focusing on the paradigm transition “from device to system,” this review systematically redefines the design rationale for MSCs in on‐chip power applications. Diverging from traditional approaches that prioritize materials or standalone performance, this review clearly establishes that the fundamental significance of MSCs resides not in their identity as high‐performance storage units, but in their systemic role as energy nodes within the microsystem architecture. Accordingly, the review introduces an integrated design framework encompassing “application requirements, system constraints, integration strategy, device architecture, and material selection,” employing it as the overarching analytical structure for the entire work. The framework's practical import resides in its ability to consolidate the formerly disparate challenges of material optimization, structural design, and fabrication process selection into a unified set of engineering decisions guided by system‐level targets. Crucially, by employing “integration strategy” as an intermediate nexus, the review delineates how system‐level requirements cascade downward to constrain device realization pathways, thus averting the research pitfall of “performance orientation with systemic incompatibility.”

Expanding on this premise, the review advances a unified analysis of monolithic and hybrid integration through the lens of functional coupling. Analyses reveal that monolithic integration achieves the lowest parasitic losses and most rapid dynamic response by reinforcing intrinsic coupling, yet its ultimate performance remains bounded by material compatibility constraints and allowable process windows. Hybrid integration, conversely, improves system flexibility via functional decoupling, albeit at the inevitable cost of increased interconnection losses and packaging intricacy. A more definitive conclusion thus emerges: the critical challenge facing current MSCs is not the attainment of “sufficiently high performance,” but rather the effective utilization of that performance within prevailing system constraints. Further analysis in the context of specific applications demonstrates that the functional role of MSCs within a system, such as power buffering, energy scheduling, voltage matching, and high‐frequency filtering, is determined not by the device itself, but conjointly by its systemic location and integration modality. This insight fundamentally illustrates that the optimal solution for MSCs design no longer resides at the discrete device level but rather lies within system‐level co‐optimization.

### 6.2 Outlook

Moving forward, the progression of MSCs from experimental devices to field‐deployable on‐chip power units depends critically not on isolated performance advances, but on the formulation of achievable integration solutions that address concrete system constraints. In light of the preceding analysis, the following avenues are identified as especially critical:

First, a unified performance evaluation standard should be established to resolve the current predicament arising from the fragmentation of performance metrics. As previously discussed, pronounced variations in measurement conditions for energy density, power density, and ESR among individual investigations severely impede cross‐comparison of experimental results and result in a dearth of parameters directly translatable to system design. Moving forward, the formulation of industry‐wide or consensus‐driven academic testing protocols is warranted, encompassing, for instance, standardized measurements of energy/power output under uniform scan rates and electrochemical windows, adoption of a comparative framework referencing MBs and thin‐film capacitors, and mandatory reporting of system‐critical parameters such as frequency response and RC time constants. The core objective is to ensure that performance data directly inform system design rather than merely showcase device‐level limits.

Second, it is essential to surmount the constraints imposed by high‐temperature fabrication processes and material incompatibilities within monolithic integration schemes. While monolithic integration theoretically affords the lowest parasitic losses, its practical implementation is circumscribed by the thermal budget limitations of CMOS BEOL processes and the intrinsic stability challenges of heterogeneous material systems. Future endeavors should prioritize the development of low‐temperature synthesis routes amenable to back‐end‐of‐line integration, alongside interface engineering strategies aimed at refining the electrode–interconnect contact junctions to minimize contact resistance. This approach would enable genuine system‐level monolithic integration without performance concessions, all while remaining compliant with the stipulated thermal budget parameters.

Third, the challenges of packaging‐induced parasitic effects and interfacial deterioration within hybrid integration schemes must be systematically resolved. Traditional encapsulation methods often impose extraneous parasitic elements and precipitate interfacial leakage along with progressive material decay, thereby critically compromising MSCs deployment in flexible electronics and biomedical applications. Corresponding strategies include the adoption of intrinsically stable solid‐state electrolytes to obviate liquid leakage hazards, coupled with the implementation of atomic layer deposition techniques to construct multilayered thin‐film barrier encapsulants that bolster overall hermeticity and shielding efficacy. Concurrently, a system‐oriented reliability evaluation paradigm is warranted, extending beyond mere device cyclability metrics, this entails, for example, assessing full MSCs module performance under diverse hygrothermal and mechanical stress regimes, thereby rectifying the prevailing research discontinuity wherein “devices excel while systems fail.”

Fourth, modularization and standardized interface protocols are imperative. These approaches can mitigate the constraints commonly seen in hybrid integration schemes, including limited system scalability and complex coupling issues. Presently, MSCs modules are largely bespoke in design, devoid of generalized electrical and packaging interconnects, thereby impeding facile scalability and expedited redeployment across diverse system platforms. Future efforts should focus on establishing unified electrical interface specifications (e.g., voltage levels, impedance matching standards) and modular package dimensions, enabling MSCs to be invoked at the system level just like other electronic components. Moreover, the co‐design of energy storage units and power management circuits should not be overlooked, for instance, developing MSCs modules compatible with prevalent printed circuit board layouts to enhance overall system integration density and scalability. By constructing standardized, electrified, and modularized MSCs components, system‐level integration efficiency can be significantly enhanced, and the design barrier substantially lowered.

Synthesizing the preceding analyses permits a prospective determination: the subsequent evolution of MSCs shall be delineated not by intrinsic material or device performance ceilings alone, but by their mode of integration and capacity for functional coupling within the microsystem environment. In other words, isolated material optimization or structural innovation is no longer sufficient to fundamentally propel MSCs toward practical application, only through the co‐design of energy storage units, interconnect structures, and functional modules under system constraints can their full potential be genuinely unleashed. Therefore, system‐level integration is not an optional trajectory for MSCs development, but rather a prerequisite for its transition from “device technology” to “system‐level energy solutions.”

## Author Contributions

**Kaiying Wang**: conceptualization, Writing – review and editing, resources, supervision, funding acquisition. **Zhuohao Liu**: investigation, writing – original draft, writing – review and editing. **Gang Li**: conceptualization, funding acquisition, writing – review and editing, supervision, resources.

## Conflicts of Interest

The authors declare no conflicts of interest.

## Data Availability

Data sharing not applicable to this article as no datasets were generated or analysed during the current study.

## References

[1] X.‐L. Li , W.‐Z. Wang , B. Li , et al., “Unique Bioinspired Nanowire Structures for Dual‐Modal Optoelectronic Electronic Skin,” Advanced Materials 37, no. 33 (2025): 2504266. 10.1002/adma.202504266 40451734

[2] Z. Ren , C. Xin , K. Liang , et al., “Femtosecond Laser Writing of Ant‐Inspired Reconfigurable Microbot Collectives,” Nature Communications 15, no. 1 (2024): 7253, 10.1038/s41467-024-51567-4. PMC11343760 39179567

[3] H. Sheng , L. Jiang , Q. Wang , et al., “A Soft Implantable Energy Supply System That Integrates Wireless Charging and Biodegradable Zn‐Ion Hybrid Supercapacitors,” Science Advances 9, no. 46 (2023): adh8083, 10.1126/sciadv.adh8083. PMC10651135 37967195

[4] J. Qin , H. Zhang , Z. Yang , et al., “Recent Advances and Key Opportunities on In‐Plane Micro‐Supercapacitors: From Functional Microdevices to Smart Integrated Microsystems,” Journal of Energy Chemistry 81 (2023): 410–431, 10.1016/j.jechem.2023.01.065.

[5] M. Pantrangi , E. Ashalley , M. K. Hadi , et al., “Flexible Micro‐Supercapacitors: Materials and Architectures for Smart Integrated Wearable and Implantable Devices,” Energy Storage Materials 73 (2024): 103791, 10.1016/j.ensm.2024.103791.

[6] F. U. Nisa , M. Tahir , S. Khalid , et al., “Revolutionizing Micro‐Scale Energy Storage by 0D Carbon Nanostructures: Synthesis, Integration, Performance Optimization Mechanisms and Sustainable Applications,” Advanced Functional Materials 35, no. 13 (2025): 2418053, 10.1002/adfm.202418053.

[7] F. Ye , W. Yang , X. Liao , C. Dong , L. Xu , and L. Mai , “A Micro Battery Supercapacitor Hybrid Device With Ultrahigh Cycle Lifespan and Power Density Enabled by Bi‐Functional Coating Design,” Advanced Functional Materials 35, no. 3 (2025): 2413379, 10.1002/adfm.202413379.

[8] A. M. Patil , A. A. Jadhav , N. R. Chodankar , et al., “Recent Progress of MXene Synthesis, Properties, Microelectrode Fabrication Techniques for Microsupercapacitors and Microbatteries Energy Storage Devices and Integration: A Comprehensive Review,” Coordination Chemistry Reviews 517 (2024): 216020, 10.1016/j.ccr.2024.216020.

[9] L. Yang , Y. Liu , R. Bi , et al., “Direct‐Ink‐Written Shape‐Programmable Micro‐Supercapacitors With Electrothermal Liquid Crystal Elastomers,” Advanced Functional Materials 35, no. 39 (2025): 2504979, 10.1002/adfm.202504979.

[10] D. B. Basha , S. Ahmed , A. Ahmed , and M. A. Gondal , “Recent Advances on Nitrogen Doped Porous Carbon Micro‐Supercapacitors: New Directions for Wearable Electronics,” Journal of Energy Storage 60 (2023): 106581, 10.1016/j.est.2022.106581.

[11] J. Wang , W. Guo , Z. Liu , and Q. Zhang , “Engineering of Self‐Aggregation‐Resistant MnO_2_ Heterostructure With A Built‐In Field for Enhanced High‐Mass‐Loading Energy Storage,” Advanced Energy Materials 13, no. 20 (2023): 2300224, 10.1002/aenm.202300224.

[12] X. Wang , W. Chen , X. Shi , et al., “Microfluidics‐Assisted Fabrication of All‐Flexible Substrate‐Free Micro‐Supercapacitors With Customizable Configuration and High Performance,” Advanced Energy Materials 13, no. 28 (2023): 2203535, 10.1002/aenm.202203535.

[13] H. Huang and W. Yang , “MXene‐Based Micro‐Supercapacitors: Ink Rheology, Microelectrode Design and Integrated System,” ACS Nano 18, no. 6 (2024): 4651–4682, 10.1021/acsnano.3c10246. 38307615

[14] H. Li , S. Ding , J. Ding , J. Luo , S. Liu , and H. Huang , “MXene‐Based Micro‐Supercapacitors Powered Integrated Sensing System: Progress and Prospects,” Energy Storage Materials 74 (2025): 103907, 10.1016/j.ensm.2024.103907.

[15] P. Zhang , S. Yang , H. Xie , et al., “Advanced Three‐Dimensional Microelectrode Architecture Design for High‐Performance On‐Chip Micro‐Supercapacitors,” ACS Nano 16, no. 11 (2022): 17593–17612, 10.1021/acsnano.2c07609. 36367555

[16] S. Wang , S. Zheng , X. Shi , et al., “Monolithically Integrated Micro‐Supercapacitors With High Areal Number Density Produced by Surface Adhesive‐Directed Electrolyte Assembly,” Nature Communications 15, no. 1 (2024): 2850, 10.1038/s41467-024-47216-5. PMC10987489 38565855

[17] R. R. Neiber , J. Kumar , B. P. Sharma , W.‐L. Ding , and X. Lu , “Ultra Stable Ink With Promising Areal Capacitance as Advanced Micro‐Supercapacitor and Unique Metal Absorptivity Enabled by Surface Functionalization of Titanium Carbide (MXene),” Advanced Functional Materials 34, no. 49 (2024): 2406481, 10.1002/adfm.202406481.

[18] H. Li and J. Liang , “Recent Development of Printed Micro‐Supercapacitors: Printable Materials, Printing Technologies, and Perspectives,” Advanced Materials 32, no. 3 (2020): 1805864, 10.1002/adma.201805864. 30941808

[19] L. Wang , Y. Ding , Z. Xu , et al., “Picosecond Ultraviolet Laser Patterned In‐Plane Asymmetric Micro‐Supercapacitors With High‐Precision Capacity Matching,” Energy Storage Materials 65 (2024): 103132, 10.1016/j.ensm.2023.103132.

[20] N. Kumar , P. K. Sahoo , S.‐Y. Lee , and S.‐J. Park , “Recent Laser Advances in Graphene‐Based Planar Micro‐Supercapacitors: Challenges and Future Prospects,” Sustainable Materials and Technologies 40 (2024): e00962, 10.1016/j.susmat.2024.e00962.

[21] J. P. Das , V. Navakoteswara Rao , and S.‐J. Kim , “Advances in Structural Engineering and Electrochemical Insights of MXene‐Based Derivates for Next Generation Micro‐Supercapacitor With Tuneable Ink, Microelectrode Design, and Scalable Manufacturing Strategies,” Progress in Materials Science 157 (2026): 101599, 10.1016/j.pmatsci.2025.101599.

[22] K. Naik , M. Saquib , S. Shetty , et al., “Recent Advances in Screen Printable Microsupercapacitors for Emerging Printed Electronics,” Journal of Energy Storage 140 (2025): 118946, 10.1016/j.est.2025.118946.

[23] K.‐H. Lee , S.‐S. Lee , D. B. Ahn , J. Lee , D. Byun , and S.‐Y. Lee , “Ultrahigh Areal Number Density Solid‐State On‐Chip Microsupercapacitors via Electrohydrodynamic Jet Printing,” Science Advances 6, no. 10 (2020): aaz1692, 10.1126/sciadv.aaz1692. PMC7060056 32181360

[24] E. Kim , J. Song , T.‐E. Song , et al., “Scalable Fabrication of MXene‐Based Flexible Micro‐Supercapacitor With Outstanding Volumetric Capacitance,” Chemical Engineering Journal 450 (2022): 138456, 10.1016/j.cej.2022.138456.

[25] Y. Zhou , J. Li , H. Fu , et al., “Additive‐Free Ti_3_C_2_T* _x_ * MXene/Carbon Nanotube Aqueous Inks Enable Energy Density Enriched 3D‐Printed Flexible Micro‐Supercapacitors for Modular Self‐Powered Systems,” Carbon Energy 7, no. 4 (2025): 698, 10.1002/cey2.698.

[26] J. Jia , Y. Zhu , P. Das , et al., “Advancing MXene‐Based Integrated Microsystems With Micro‐Supercapacitors and/or Sensors: Rational Design, Key Progress, and Challenging Perspectives,” Journal of Materiomics 9, no. 6 (2023): 1242–1262, 10.1016/j.jmat.2023.08.013.

[27] H.‐W. Li , J. Piwek , and A. M. Ionescu , “Nitrogen Doping of Vertically Aligned Carbon Nanotubes for On‐Chip CMOS‐Compatible Pseudocapacitive Supercapacitors,” Carbon 247 (2026): 121018, 10.1016/j.carbon.2025.121018.

[28] F. Li , Y. Li , J. Qu , et al., “Recent Developments of Stamped Planar Micro‐Supercapacitors: Materials, Fabrication and Perspectives,” Nano Materials Science 3, no. 2 (2021): 154–169, 10.1016/j.nanoms.2020.10.003.

[29] S. Wang , J. Ma , X. Shi , Y. Zhu , and Z.‐S. Wu , “Recent Status and Future Perspectives of Ultracompact and Customizable Micro‐Supercapacitors,” Nano Research Energy 1 (2022): 9120018, 10.26599/NRE.2022.9120018.

[30] M. Saqib , A. Mannan , M. Noman , et al., “Miniaturizing Power: Harnessing Micro‐Supercapacitors for Advanced Micro‐Electronics,” Chemical Engineering Journal 490 (2024): 151857, 10.1016/j.cej.2024.151857.

[31] S. K. Hong , C. S. Kim , W. S. Hwang , and B. J. Cho , “Hybrid Integration of Graphene Analog and Silicon Complementary Metal–Oxide–Semiconductor Digital Circuits,” ACS Nano 10, no. 7 (2016): 7142–7146, 10.1021/acsnano.6b03382. 27403730

[32] J. H. Kim , S. Aghaeimeibodi , J. Carolan , D. Englund , and E. Waks , “Hybrid Integration Methods for On‐Chip Quantum Photonics,” Optica 7, no. 4 (2019): 291–308, 10.1364/OPTICA.384118.

[33] A. C. Fischer , F. Forsberg , M. Lapisa , et al., “Integrating MEMS and ICs,” Microsystems & Nanoengineering 1, no. 1 (2015): 15005.

[34] S. Wang , L. Li , S. Zheng , et al., “Monolithic Integrated Micro‐Supercapacitors With Ultra‐High Systemic Volumetric Performance and Areal Output Voltage,” National Science Review 10, no. 3 (2023): nwac271, 10.1093/nsr/nwac271. 36875784 PMC9976746

[35] A. C. Paolella , R. DeSalvo , C. Middleton , et al., “Hybrid Integration of RF Photonic Systems,” Journal of Lightwave Technology 36, no. 21 (2018): 5067–5073, 10.1109/JLT.2018.2870252.

[36] N. A. Kyeremateng , T. Brousse , and D. Pech , “Microsupercapacitors as Miniaturized Energy‐Storage Components for On‐Chip Electronics,” Nature Nanotechnology 12, no. 1 (2017): 7–15, 10.1038/nnano.2016.196. 27819693

[37] M. Yuan , F. Luo , Z. Wang , J. Yu , H. Li , and X. Chen , “Smart Wearable Band‐Aid Integrated With High‐Performance Micro‐Supercapacitor, Humidity and Pressure Sensor for Multifunctional Monitoring,” Chemical Engineering Journal 453 (2023): 139898, 10.1016/j.cej.2022.139898.

[38] G. Wu , S. Sun , X. Zhu , Z. Ma , Y. Zhang , and N. Bao , “Microfluidic Fabrication of Hierarchical‐Ordered ZIF‐L(Zn)@Ti_3_C_2_T* _x_ * Core–Sheath Fibers for High‐Performance Asymmetric Supercapacitors,” Angewandte Chemie 134, no. 8 (2022): 202115559, 10.1002/ange.202115559. 34919307

[39] S. S. Nardekar and S.‐J. Kim , “Untethered Magnetic Soft Robot With Ultra‐Flexible Wirelessly Rechargeable Micro‐Supercapacitor as an Onboard Power Source,” Advanced Science 10, no. 28 (2023): 2303918, 10.1002/advs.202303918. 37544914 PMC10558651

[40] X. Feng , S. Wang , P. Das , et al., “Ultrahigh‐Rate and High‐Frequency MXene Micro‐Supercapacitors for kHz AC Line‐Filtering,” Journal of Energy Chemistry 69 (2022): 1–8, 10.1016/j.jechem.2021.11.012.

[41] W. Wang , L. Xu , L. Zhang , A. Zhang , and J. Zhang , “Self‐Powered Integrated Sensing System With In‐Plane Micro‐Supercapacitors for Wearable Electronics,” Small 19, no. 29 (2023): 2207723, 10.1002/smll.202207723. 37046182

[42] S. Chen , Z. Li , P.‐H. Huang , et al., “Ultrafast Metal‐Free Microsupercapacitor Arrays Directly Store Instantaneous High‐Voltage Electricity From Mechanical Energy Harvesters,” Advanced Science 11, no. 22 (2024): 2400697, 10.1002/advs.202400697. 38502870 PMC11165484

[43] X. Lin , S. Li , X. Li , et al., “3D Patterned Fabric‐Based Wearable Micro‐Supercapacitor Operating at High Voltage by Electrostatic Actuation,” npj Flexible Electron 9, no. 1 (2025): 60.

[44] S. Rani , G. Khandelwal , S. Kumar , et al., “Flexible Self‐Powered Supercapacitors Integrated With Triboelectric Nanogenerators,” Energy Storage Materials 74 (2025): 103977, 10.1016/j.ensm.2024.103977.

[45] S. A. Adewinbi , B. A. Taleatu , M. Roble , F. Vega , and D. Diaz‐Droguett , “Developments on Flexible Micro‐Supercapacitor Electrodes: From the Basics to Functional Photo‐Charging Integrated Power Systems,” Journal of Energy Storage 101 (2024): 113885, 10.1016/j.est.2024.113885.

[46] L. Wang , H. Wang , C. Wu , et al., “Moisture‐Enabled Self‐Charging and Voltage Stabilizing Supercapacitor,” Nature Communications 15, no. 1 (2024): 4929, 10.1038/s41467-024-49393-9. PMC11165001 38858397

[47] M. F. El‐Kady and R. B. Kaner , “Scalable Fabrication of High‐Power Graphene Micro‐Supercapacitors for Flexible and On‐Chip Energy Storage,” Nature Communications 4, no. 1 (2013): 1475, 10.1038/ncomms2446. 23403576

[48] Z. Ren , X. Shi , Q. Yang , et al., “An Ultrastretchable Seamlessly Integrated Contactless Charging Microsystem Towards Skin‐Attachable Wireless Microelectronics,” Nature Communications 16, no. 1 (2025): 1642, 10.1038/s41467-025-56881-z. PMC11828911 39952967

[49] K. Manojkumar , M. Muthuramalingam , A. Sundamoorthy , et al., “Revolutionizing Energy Storage: Self‐Charging Supercapacitors Toward Self‐Powered Micro and Nano Systems,” Chemical Engineering Journal 524 (2025): 168642, 10.1016/j.cej.2025.168642.

[50] S. L. Kim , H. T. Lin , and C. Yu , “Thermally Chargeable Solid‐State Supercapacitor,” Advanced Energy Materials 6, no. 18 (2016): 1600546, 10.1002/aenm.201600546.

[51] G. L. Park , A. I. Schäfer , and B. S. Richards , “Renewable Energy‐Powered Membrane Technology: Supercapacitors for Buffering Resource Fluctuations in a Wind‐Powered Membrane System for Brackish Water Desalination,” Renewable Energy 50 (2013): 126–135, 10.1016/j.renene.2012.05.026.

[52] Z. Cabrane , J. Kim , K. Yoo , and M. Ouassaid , “HESS‐Based Photovoltaic/Batteries/Supercapacitors: Energy Management Strategy and DC Bus Voltage Stabilization,” Solar Energy 216 (2021): 551–563, 10.1016/j.solener.2021.01.048.

[53] X. Fu , X. Pan , Y. Liu , et al., “Non‐Contact Triboelectric Nanogenerator,” Advanced Functional Materials 33, no. 52 (2023): 2306749.

[54] D. Chen , R. Li , J. Xu , D. Li , C. Fei , and Y. Yang , “Recent Progress and Development of Radio Frequency Energy Harvesting Devices and Circuits,” Nano Energy 117 (2023): 108845, 10.1016/j.nanoen.2023.108845.

[55] M. Zhu , M. Hassanalieragh , Z. Chen , A. Fahad , K. Shen , and T. Soyata , “Energy‐Aware Sensing in Data‐Intensive Field Systems Using Supercapacitor Energy Buffer,” IEEE Sensors Journal 18, no. 8 (2018): 3372–3383, 10.1109/JSEN.2018.2809683.

[56] A. Hosseini , J. Adabi , S. A. Gholamian , and S. Y. Mousazadeh Mousavi , “Improving the Performance of Supercapacitor‐Based Pulsed Power Systems in DC Microgrids Using a Fast Integral Terminal Super‐Twisting Sliding Mode Controller,” Journal of Energy Storage 153 (2026): 120660, 10.1016/j.est.2026.120660.

[57] X. Li , Y. Yang , J. Zhang , P. Li , F. Guo , and H. Zhang , “A Hierarchical Real‐Time Energy Management and Control Strategy for Fully‐Active Battery/Supercapacitor Hybrid Energy Storage System,” Journal of Energy Storage 141 (2026): 119384, 10.1016/j.est.2025.119384.

[58] A. C R , U. B. Manthati , and S. Punna , “Supercapacitor Voltage Based Power Sharing and Energy Management Strategy for Hybrid Energy Storage System,” Journal of Energy Storage 50 (2022): 104232, 10.1016/j.est.2022.104232.

[59] H. Mahmood and J. Jiang , “Decentralized Power Management of Multiple PV, Battery, and Droop Units in an Islanded Microgrid,” IEEE Transactions on Smart Grid 10, no. 2 (2019): 1898–1906, 10.1109/TSG.2017.2781468.

[60] S. Fang , S. Hu , Y. Liu , C. Zhao , and Y. Wang , “Power Management Unit With Maximum‐Efficiency‐Point‐Tracking to Enhance the Efficiency of Micro DMFC Stack,” Energy 315 (2025): 134353.

[61] C. W. Liu and L. R. Chang‐Chien , “Area Efficient High‐Performance Digitally Controlled Power Management Unit,” IEEE Transactions on Industrial Electronics 68, no. 3 (2021): 2437–2446, 10.1109/TIE.2020.2975476.

[62] P. Mayer , M. Magno , and L. Benini , “Smart Power Unit—mW‐to‐nW Power Management and Control for Self‐Sustainable IoT Devices,” IEEE Transactions on Power Electronics 36, no. 5 (2021): 5700–5710, 10.1109/TPEL.2020.3031697.

[63] J. Li , B. Han , X. Tian , W. Bao , and C. Henry , “Vehicle Peak Power Management System: Design, Development, and Testing of a Fuel Cell and Supercapacitor Hybrid,” Case Studies in Thermal Engineering 75 (2025): 107178.

[64] A. Cano , P. Arévalo , and F. Jurado , “Evaluation of Temporal Resolution Impact on Power Fluctuations and Self‐Consumption for a Hydrokinetic on Grid System Using Supercapacitors,” Renewable Energy 193 (2022): 843–856, 10.1016/j.renene.2022.05.070.

[65] J. Pegueroles‐Queralt , F. D. Bianchi , and O. Gomis‐Bellmunt , “A Power Smoothing System Based on Supercapacitors for Renewable Distributed Generation,” IEEE Transactions on Industrial Electronics 62, no. 1 (2015): 343–350, 10.1109/TIE.2014.2327554.

[66] Q. Zhou , Z. Yang , Z. Cao , et al., “Controllable Carbon Shell Encapsulation via Rapid Joule Heating Calcination for High‐Performance Asymmetric Supercapacitor With Suppressed Self‐Discharge and Robust Cycling Stability,” Advanced Science (2026): 76184, 10.1002/advs.76184. PMC13336865 42312396

[67] L. Song , W. Hou , H. Qiao , et al., “Conquering Self‐Discharge in Supercapacitors: Synergy of Mechanisms and Cross‐Scale Mitigation Strategies,” Journal of Materials Chemistry A 14, no. 21 (2026): 12479–12508, 10.1039/D5TA08045B.

[68] W. Shang , W. Yu , X. Xiao , et al., “Insight Into the Self‐Discharge Suppression of Electrochemical Capacitors: Progress and Challenges,” Advanced Powder Materials 2, no. 1 (2023): 100075, 10.1016/j.apmate.2022.100075.

[69] R. German , A. Sari , O. Briat , J. M. Vinassa , and P. Venet , “Impact of Voltage Resets on Supercapacitors Aging,” IEEE Transactions on Industrial Electronics 63, no. 12 (2016): 7703–7711, 10.1109/TIE.2016.2594786.

[70] C. Luo , J. Zhao , S. Ma , et al., “Fast Two‐Stage Charge Equaliser Based on State‐of‐Charge (SOC) Balancing for Series‐Connected Supercapacitors,” The Journal of Engineering 2019, no. 16 (2019): 2615–2620, 10.1049/joe.2018.8565.

[71] H. Ahmad Abubakar , D. Isa , and W. Y. Wan , “Comparing the Degradation Effect of a ‘Two‐Cell’ Supercapacitor‐Module With and Without Voltage Equalization Circuit(s) Under Experimental Self‐Discharge and Load Cycling Tests,” Microelectronics Reliability 79 (2017): 140–148, 10.1016/j.microrel.2017.10.025.

[72] K. Mongkoldee , E. Sukjit , and T. Kulworawanichpong , “Optimal On‐Board Energy Buffer Design for Fuel‐Cell Hybrid Intercity Vehicles,” Journal of Energy Storage 46 (2022): 103820, 10.1016/j.est.2021.103820.

[73] J. Yim , S. You , F. Blaabjerg , Y. Lee , Y. Gui , and W. Kim , “Energy Management Systems for Forecasted Demand Error Compensation Using Hybrid Energy Storage System in Nanogrid,” Renewable Energy 221 (2024): 119744, 10.1016/j.renene.2023.119744.

[74] A. M. A. Malkawi , M. I. Alawneh , and A. Bashaireh , “A Seamless Start‐Up for a Hybrid Uninterruptible Power Supply Based on a Diesel Generator and Supercapacitor Energy Storage,” Results in Engineering 24 (2024): 103418, 10.1016/j.rineng.2024.103418.

[75] J. Chen , D. Han , J. Deng , et al., “Unlocking Maximum Synergy: Screen‐Printing Fabrication of Heterostructured Microsupercapacitor Stacks,” Small Methods 8, no. 9 (2024): 2301506, 10.1002/smtd.202301506. 38752313

[76] S. Xu , F. Xia , Z. Li , et al., “Wafer‐Level Heterogeneous Integration of Electrochemical Devices and Semiconductors for a Monolithic Chip,” National Science Review 11, no. 10 (2024): nwae049, 10.1093/nsr/nwae049. 39301075 PMC11409884

[77] P. Huang , C. Lethien , S. Pinaud , et al., “On‐Chip and Freestanding Elastic Carbon Films for Micro‐Supercapacitors,” Science 351, no. 6274 (2016): 691–695, 10.1126/science.aad3345. 26912855

[78] F. Dastageer and A. S. Areeckal , “On‐Chip Integration of Micro‐Supercapacitor in VLSI Design for Power Management in Artificial Intelligence Processors and Memory Chips: A Review of Methods and Materials,” Arabian Journal for Science and Engineering 50, no. 19 (2025): 15219–15234.

[79] S. Jeon , H. Seo , Y. Kim , et al., “Fully Biocompatible, Thermally Drawn Fiber Supercapacitors for Long‐Term Bio‐Implantation,” Nature Communications 16, no. 1 (2025): 8207, 10.1038/s41467-025-63649-y. PMC12405512 40897716

[80] R. Schneider , T. Katsila , M. Jafarpour , et al., “Towards Implantable Supercapacitors With High Longevity,” Journal of Energy Storage 141 (2026): 119204, 10.1016/j.est.2025.119204.

[81] Z. Luo , B. Kou , Y. Wang , et al., “Toy‐Blocks‐Inspired Programmable Supercapacitors With High Energy Density,” Chemical Engineering Journal 445 (2022): 136788, 10.1016/j.cej.2022.136788.

[82] B. Lu , F. Liu , G. Sun , et al., “Compact Assembly and Programmable Integration of Supercapacitors,” Advanced Materials 32, no. 6 (2020): 1907005. 10.1002/adma.201907005 31850657

[83] M. Beidaghi and Y. Gogotsi , “Capacitive Energy Storage in Micro‐Scale Devices: Recent Advances in Design and Fabrication of Micro‐Supercapacitors,” Energy & Environmental Science 7, no. 3 (2014): 867–884, 10.1039/c3ee43526a.

[84] D. Li , S. Yang , X. Chen , W.‐Y. Lai , and W. Huang , “3D Wearable Fabric‐Based Micro‐Supercapacitors With Ultra‐High Areal Capacitance,” Advanced Functional Materials 31, no. 50 (2021): 2107484, 10.1002/adfm.202107484.

[85] Q. He , Z.‐C. Ling , D.‐H. Li , et al., “Sargassum Nanocellulose‐Based Fully Ingestible Supercapacitor,” Advanced Materials 37, no. 9 (2025): 2416307. 10.1002/adma.202416307 39838771

[86] Q. Lv , X. Li , X. Tian , et al., “A Degradable and Biocompatible Supercapacitor Implant Based on Functional Sericin Hydrogel Electrode,” Advanced Energy Materials 13, no. 16 (2023): 2203814.

[87] S. Xu , W. Liu , B. Hu , and X. Wang , “Circuit‐Integratable High‐Frequency Micro Supercapacitors With Filter/Oscillator Demonstrations,” Nano Energy 58 (2019): 803–810, 10.1016/j.nanoen.2019.01.079.

[88] X. Feng , X. Shi , J. Ning , et al., “Recent Advances in Micro‐Supercapacitors for AC Line‐Filtering Performance: From Fundamental Models to Emerging Applications,” eScience 1, no. 2 (2021): 124–140.

[89] Y. Hu , M. Wu , F. Chi , et al., “Ultralow‐Resistance Electrochemical Capacitor for Integrable Line Filtering,” Nature 624, no. 7990 (2023): 74–79, 10.1038/s41586-023-06712-2. 37968404

[90] W. Li , X. Yang , Z. Chen , T. Lv , X. Wang , and J. Qiu , “Synthesis and Structure Regulation of Armor‐Wearing Biomass‐Based Porous Carbon: Suppression the Leakage Current and Self‐Discharge of Supercapacitors,” Carbon 196 (2022): 136–145, 10.1016/j.carbon.2022.04.037.

[91] C. Gao , Q. You , J. Huang , et al., “Ultraconformable Integrated Wireless Charging Micro‐Supercapacitor Skin,” Nano‐Micro Letters 16, no. 1 (2024): 123, 10.1007/s40820-024-01352-1. 38372847 PMC10876509

[92] M. K. Hota , Q. Jiang , Z. Wang , Z. L. Wang , K. N. Salama , and H. N. Alshareef , “Integration of Electrochemical Microsupercapacitors With Thin Film Electronics for On‐Chip Energy Storage,” Advanced Materials 31, no. 25 (2019): 1807450, 10.1002/adma.201807450. 31058380

[93] N. Sani , U. Linderhed , and M. Sandberg , “Monolithically Integrated Electrochemical Energy Storage Modules,” Journal of Energy Storage 16 (2018): 139–144, 10.1016/j.est.2018.01.004.

[94] K.‐H. Lee , S.‐W. Kim , M. Kim , et al., “Folding the Energy Storage: Beyond the Limit of Areal Energy Density of Micro‐Supercapacitors,” Advanced Energy Materials 13, no. 20 (2023): 2204327.

[95] Y. Xie , H. Zhang , X. Jiang , et al., “In‐Situ Construction of Integrated Asymmetric Micro‐Supercapacitors Achieving Monolithic Hundred‐Volt Output,” Journal of Colloid and Interface Science 677 (2025): 12–20, 10.1016/j.jcis.2024.07.249. 39128197

[96] L. Zhang , J. Qin , P. Das , et al., “Electrochemically Exfoliated Graphene Additive‐Free Inks for 3D Printing Customizable Monolithic Integrated Micro‐Supercapacitors on a Large Scale,” Advanced Materials 36, no. 19 (2024): 2313930, 10.1002/adma.202313930. 38325888

[97] Y. Yuan , X. Li , L. Jiang , et al., “Laser Maskless Fast Patterning for Multitype Microsupercapacitors,” Nature Communications 14, no. 1 (2023): 3967, 10.1038/s41467-023-39760-3. PMC10322851 37407565

[98] G. Saeed , T. Kang , J. S. Byun , et al., “Two‐Dimensional (2D) Materials for 3D Printed Micro‐Supercapacitors and Micro‐Batteries,” Energy Mater 4, no. 3 (2024): 400023.

[99] Y. Shao , J.‐H. Fu , Z. Cao , et al., “3D Crumpled Ultrathin 1T MoS_2_ for Inkjet Printing of Mg‐Ion Asymmetric Micro‐Supercapacitors,” ACS Nano 14, no. 6 (2020): 7308–7318, 10.1021/acsnano.0c02585. 32478507 PMC7467814

[100] C.‐Y. Ren , S.‐Y. Qiu , J.‐R. Zhai , et al., “Ionic Liquid‐Wrapped MXene Film With Bowl‐Like Structures for Highly Integrated Micro‐Supercapacitor Array With Ultrahigh Output Voltage,” Nano Research 16, no. 4 (2023): 4926–4932, 10.1007/s12274-022-5098-4.

[101] Y. Wang , Y. Zhao , Y. Han , et al., “Fixture‐Free Omnidirectional Prestretching Fabrication and Integration of Crumpled In‐Plane Micro‐Supercapacitors,” Science Advances 8, no. 21 (2022): abn8338, 10.1126/sciadv.abn8338. PMC9140961 35622921

[102] Z. Li , S. Chen , Y. Fu , and J. Li , “Efficiency Optimization for Large‐Scale Droplet‐Based Electricity Generator Arrays With Integrated Microsupercapacitor Arrays,” Nature Communications 16, no. 1 (2025): 8530, 10.1038/s41467-025-64289-y. PMC12475142 41006330

[103] J. Ma , Y. Li , Z. Wang , et al., “2D Ultrathin Graphene Heterostructures for Printable High‐Energy Micro‐Supercapacitors Integrated Into Coplanar Flexible All‐in‐One Microelectronics,” Materials Today 74 (2024): 58–66.

[104] P. Zhao , X. Mao , J. Song , et al., “Integrated Self‐Powered Sensors for Continuous Foot Health Monitoring via Laser‐Induced MXene‐Composited Graphene Hybrids From Lignocellulose,” Advanced Science 13, no. 2 (2026): 16691, 10.1002/advs.202516691. PMC12786368 41124659

[105] D. Das Sharma , G. Pasdast , S. Tiagaraj , and K. Aygün , “High‐Performance, Power‐Efficient Three‐Dimensional System‐in‐Package Designs With Universal Chiplet Interconnect Express,” Nature Electronics 7, no. 3 (2024): 244–254.

[106] J. Fan , C. Lou , P. Cui , et al., “A Wearable Self‐Charging Power System Integrating Micro‐Supercapacitors and Triboelectric Nanogenerators With MXene‐Coated Fabric as Conductive Layer,” Advanced Powder Materials 4, no. 6 (2025): 100341, 10.1016/j.apmate.2025.100341.

[107] D. Ren , S. Zhang , J. Dai , et al., “Sulfur‐Functionalized Carbon Nanotubes With Inlaid Nanographene for 3D‐Printing Micro‐Supercapacitors and a Flexible Self‐Powered Sensing System,” ACS Nano 18, no. 31 (2024): 20706–20715, 10.1021/acsnano.4c06879. 39051159

[108] S. Zampolli , I. Elmi , P. Bruschi , et al., “An ASIC‐Based System‐in‐Package MEMS Gas Sensor With Impedance Spectroscopy Readout and AI‐Enabled Identification Capabilities,” Sensors and Actuators B: Chemical 424 (2025): 136924.

[109] M. Schuenemann , V. Grosser , R. Leutenbauer , G. Bauer , W. Schaefer , and H. Reichl , “A Highly Flexible Design and Production Framework for Modularized Microelectromechanical Systems,” Sensors and Actuators A: Physical 73, no. 1 (1999): 153–168.

[110] A. Wu , L. Wang , E. Jensen , R. Mathies , and B. Boser , “Modular Integration of Electronics and Microfluidic Systems Using Flexible Printed Circuit Boards,” Lab on A Chip 10, no. 4 (2010): 519–521, 10.1039/B922830F. 20126694

[111] Y. Yuan , W. Yuan , Y. Wu , et al., “High‐Performance All‐Printed Flexible Micro‐Supercapacitors With Hierarchical Encapsulation,” Energy & Environmental Materials 7, no. 4 (2024): 12657, 10.1002/eem2.12657.

[112] Y. Liu , T. Zou , C.‐Y. Xu , L. Zhen , Y. Li , and Y.‐Y. Noh , “Atomic Layer Deposition in Transistors and Monolithic 3D Integration,” Advanced Functional Materials 36, no. 41 (2026): 74729, 10.1002/adfm.74729.

[113] K.‐W. Kim , S. J. Park , S.‐J. Park , et al., “Deformable Micro‐Supercapacitor Fabricated via Laser Ablation Patterning of Graphene/Liquid Metal,” npj Flexible Electron 8, no. 1 (2024): 18.

[114] L. Li , H. Lin , S. Qiao , et al., “Monolithically Integrated Stretchable Photonics,” Light: Science & Applications 7, no. 2 (2018): 17138. 10.1038/lsa.2017.138 PMC6060064 30839545

[115] J. Wang , F. Li , F. Zhu , and O. G. Schmidt , “Recent Progress in Micro‐Supercapacitor Design, Integration, and Functionalization,” Small Methods 3, no. 8 (2019): 1800367, 10.1002/smtd.201800367.

[116] Z. Chen , C. Yan , Q. Dang , Y. Wang , Y. Xu , and X. Deng , “High‐Performance Planar Micro‐Supercapacitor Arrays for Integrated Photo‐Detecting Systems,” Science China Materials 67, no. 7 (2024): 2169–2181, 10.1007/s40843-023-2877-x.

[117] S. Ding , Q. Wang , Q. Wang , and H. Li , “Micro‐supercapacitors for Smart Transportation and Low‐Altitude Economy Applications: Advances in Material Innovations, Device Architectures, and System Integration,” Journal of Materials Chemistry A 14, no. 20 (2026): 11815–11830, 10.1039/D5TA09042C.

[118] J. S. Fandiño , P. Muñoz , D. Doménech , and J. Capmany , “A Monolithic Integrated Photonic Microwave Filter,” Nature Photonics 11, no. 2 (2017): 124–129.

[119] A. Shakoor , B. C. Cheah , D. Hao , et al., “Plasmonic Sensor Monolithically Integrated With a CMOS Photodiode,” ACS Photonics 3, no. 10 (2016): 1926–1933.

[120] K. H. Li , H. Lu , W. Y. Fu , Y. F. Cheung , and H. W. Choi , “Intensity‐Stabilized LEDs With Monolithically Integrated Photodetectors,” IEEE Transactions on Industrial Electronics 66, no. 9 (2019): 7426–7432, 10.1109/TIE.2018.2873522.

[121] R. Tao , Y. Mao , C. Gu , and W. Hu , “Integrating All‐Yarn‐Based Triboelectric Nanogenerator/Supercapacitor for Energy Harvesting, Storage and Sensing,” Chemical Engineering Journal 496 (2024): 154358, 10.1016/j.cej.2024.154358.

[122] Y. Gao , M. Rezaie , and S. A. Choi , “A Wearable, Disposable Paper‐Based Self‐Charging Power System Integrating Sweat‐Driven Microbial Energy Harvesting and Energy Storage Devices,” Nano Energy 104 (2022): 107923, 10.1016/j.nanoen.2022.107923.

[123] X. Gao , Y. Zhang , S. Yin , et al., “Dual Redox Active Sites N‐C@Ni_2_P/NiSe_2_ Heterostructure Supercapacitor Integrated With Triboelectric Nanogenerator Toward Efficient Energy Harvesting and Storage,” Advanced Functional Materials 32, no. 38 (2022): 2204833, 10.1002/adfm.202204833.

[124] Y. Zhao , M. Luo , Y. Hu , et al., “Laser Micro/Nano Fabrication of Electrochemical Filtering Capacitors: Multiscale Structural Engineering for High‐Frequency Power Conditioning,” Advanced Energy Materials 15, no. 48 (2025): 02620, 10.1002/aenm.202502620.

[125] J. Park and W. Kim , “History and Perspectives on Ultrafast Supercapacitors for AC Line Filtering,” Advanced Energy Materials 11, no. 27 (2021): 2003306, 10.1002/aenm.202003306.

[126] C. Chen , J. Cao , X. Wang , et al., “Highly Stretchable Integrated System for Micro‐Supercapacitor With AC Line Filtering and UV Detector,” Nano Energy 42 (2017): 187–194, 10.1016/j.nanoen.2017.10.056.

[127] Y. Xie , S. Pu , Z. Wang , X. Zhang , A. Cabot , and H. Zhang , “2D‐Supported Vertical MXenes for Ultrafast Filtering With Ultralow Inductance,” Nano Letters 25, no. 19 (2025): 7867–7874, 10.1021/acs.nanolett.5c01020. 40310880

